# l-Rhamnose from Seaweeds, Microalgae, and Cyanobacteria: Sourcing, Production, and Bioprocessing Strategies

**DOI:** 10.3390/md24100331

**Published:** 2026-09-22

**Authors:** Thomas Flahaut, Pascal Dubessay, Elodie Peghaire, Gwendoline Christophe, Alina Violeta Ursu, Philippe Michaud, Guillaume Pierre

**Affiliations:** 1 Université Clermont Auvergne, Clermont Auvergne INP, CNRS, Institut Pascal, 63000 Clermont-Ferrand, France; thomas.flahaut@doctorant.uca.fr (T.F.); pascal.dubessay@uca.fr (P.D.); elodie.peghaire@uca.fr (E.P.); gwendoline.christophe@uca.fr (G.C.); alina_violeta.ursu@uca.fr (A.V.U.); philippe.michaud@uca.fr (P.M.); 2 Institut Universitaire de France (IUF), 1 Rue Descartes, 75005 Paris, France

**Keywords:** rhamnose, macroalgae, microalgae, cyanobacteria, polysaccharides, process

## Abstract

l-Rhamnose (6-deoxy-l-mannose) is a rare deoxyhexose recognized for its significant value in the food, pharmaceutical and nutraceutical industries due to its unique physiological effects. While primarily utilized as a reagent in organic synthesis, l-Rha and l-Rha-enriched fractions are conventionally sourced from terrestrial plants (fruits, buds, leaves, barks, and stems) and bacteria (including *Klebsiella* sp., *Pseudomonas aeruginosa*, and *Mycobacterium* spp.). Yet, l-Rha co-extraction often leads to contamination with other monosaccharides or aglycone compounds prior to hydrolysis, which is typically carried out using chemical and/or enzymatic methods. Furthermore, the purification steps necessary to obtain high-purity l-Rah generally involve organic solvents and long separation processes that are poorly suited for large-scale applications. Future biotechnological processes must therefore be critically re-evaluated through intelligent sourcing, specifically tailored downstream processing technologies (particularly for purification), and innovative chemo-enzymatic approaches to boost l-Rha production. This review provides a comprehensive overview of current aquatic sources, structural features, production processes, and properties of l-Rha and l-Rha-enriched fractions. Emphasis is placed on novel insights into l-Rha-enriched polysaccharides produced by macroalgae, microalgae, and bacteria. Finally, perspectives on next-generation bioprocessing strategies and key challenges affecting the future of efficient l-Rha production are discussed.

## 1. Introduction

l-Rhamnose is a rare natural sugar, defined either as a 6-deoxy-hexose or as a 5-methyl-pentose. Unlike most naturally occurring sugars, which belong to the d-series, it has the distinctive feature of being an l-isomer. It is found in plants, bacteria [1], fungi [2], and viruses [3], but it is not synthesized by mammals [4]. In plants and bacteria, rhamnose does not occur in free form; instead, it is present as a nucleotide-diphosphate sugar, i.e., uridine 5′-diphospho-β-l-rhamnose (UDP-rhamnose) in plants and deoxythymidine 5′-diphospho-β-l-rhamnose (dTDP-rhamnose) in bacteria. UDP-rhamnose and dTDP-rhamnose serve as glycosyl donors for the transfer of rhamnose into rhamnose-containing molecules through the action of rhamnosyltransferases, which are part of the glycosyltransferase (GT) family. l-rhamnose biosynthesis generally starts with UDP-glucose in plants and dTDP-glucose in bacteria (Figure 1). The pathway involves three steps: dehydration of glucose to form 4-keto-6-deoxy-glucose, epimerization to produce 4-keto-l-rhamnose, and reduction to yield rhamnose. Plants can synthesize rhamnose with a single multifunctional enzyme, rhamnose synthase (RHM), which combines all three activities, or alternatively through two enzymes: a UDP-α-d-glucose 4,6-dehydratase and a bifunctional nucleotide-rhamnose synthase/epimerase-reductase. In bacteria, three distinct enzymes are required: RmlB, RmlC and RmlD [5,6]. Furthermore, a fusion protein of RmlC and RmlD has been identified in certain algal species, notably within the Haptophyta and Gymnodiniaceae families of microalgae. In *Prymnesium parvum*, the enzyme is capable of synthesizing dTDP-β-l-rhamnose or UDP-β-l-rhamnose [6].

l-rhamnose is used in the synthesis of organic compounds and as a structural component of rhamnose-containing molecules across a wide range of applications. In the agrifood industry, l-rhamnose is used as a precursor in the synthesis of aromas and flavors; indeed, rhamnose and fucose/proline/glycerol systems generate 2-propionyl-1(3),4,5,6-tetrahydropyridines (popcorn aroma) [7,8]. Rhamnose is also used to produce 4-hydroxy-2,5-dimethyl-3(2H)-furanone (caramel aroma) or 5-methyl-2-furfurylthiol (coffee aroma) via the Maillard reaction [9]. In cosmetics, its physicochemical properties allow its use as a humectant or fragrance ingredient [10]. Moreover, rhamnose-rich oligo- and polysaccharides can counteract several mechanisms involved in skin aging, including the stimulation of cell proliferation and collagen biosynthesis, the decrease in elastase-type activity, and the protection of hyaluronan against free radicals. This action is mediated by a specific lectin site recognizing α-l-rhamnose [11]. l-rhamnose is also a constituent of rhamnolipids, which are biosurfactants widely used in products such as acne pads, anti-dandruff formulations, deodorants, nail care products, toothpastes, and shampoos, where they can substitute for traditional petrochemical-based surfactants [12]. In pharmacology, rhamnose is of particular interest in cancer treatment [13,14,15]. It can be used as an inducer for recombinant proteins with the *rhaBAD* promoter [16]. Moreover, because humans cannot synthesize rhamnose, both the sugar and its biosynthetic pathway represent promising targets for novel antibiotics, as rhamnose is a key component of the cell wall, capsule, or *O*-antigen in several pathogenic bacteria [1,17]. Rhamnose metabolism could also be targeted for the development of fungicides, since in fungi, rhamnose-containing glycans play an essential role in host–pathogen interactions [2,18].

The global production of l-rhamnose remains difficult to quantify accurately due to the limited availability of publicly accessible data and the lack of comprehensive industrial production statistics. Nevertheless, commercial market estimates suggest that the annual market volume may have reached approximately 720 tons in 2025, with an average price of USD 46/kg [19].

Current commercial sources of rhamnose are primarily derived from plants such as *Sophora japonica* [20] and citrus species [21]. Plant-derived rhamnose-containing polysaccharides include hemicellulose [22], gum arabic [23], pectin, rhamnogalacturonan I and II [24], α-rhamnan [25], and ulvan [26]. The production of l-rhamnose through fermentation has also been reported for the genus *Klebsiella*, which produces rhamnose-rich exopolysaccharides (EPS) [27]. EPS are polysaccharides secreted extracellularly. Other microbial polysaccharides containing rhamnose include sphingan from the genus *Sphingomonas*, such as welan, rhamsan, diutan, and gellan gum [28]. Yet, the use of such polysaccharides is challenged by contamination with other monosaccharides, making purification and hydrolysis steps necessary. In microbial cultures, EPS accumulation also increases the viscosity of the culture medium, thereby impeding oxygen transfer, mixing, and downstream separation. In addition, polysaccharides are often contaminated with proteins, requiring further purification steps [21]. Another source of rhamnose is rhamnosides, a class of glycosides containing rhamnose. l-Rhamnose can be obtained by the hydrolysis of quercitrin (oak bark), naringin (citrus peels), and rutin (oak bark, buckwheat). These processes typically generate toxic, corrosive, and aromatic by-products [21]. Acid hydrolysis of rutin extracted from *Sophora japonica* buds is also a possible source [20]. In bacteria, rhamnolipids produced by *Pseudomonas aeruginosa* can serve as a rhamnose source [21], although their exploitation is limited by the pathogenicity of the producer strain. In both plant polysaccharides and rhamnosides, the use of terrestrial biomass requires arable land and involves the transportation and processing of large amounts of raw material, generating significant quantities of by-products. These constraints, combined with increasing market demand, highlight the need for alternative sources of rhamnose.

Terrestrial plants have been extensively investigated for l-Rha production, particularly regarding rhamnogalacturonans and rhamnose-containing glycosides; indeed, a previous review synthesized the available information on dicotyledonous and grass (commelinid monocotyledons) species [5]. However, no review has specifically examined and evaluated the potential of aquatic organisms, leaving a significant and unexplored area of alternative sources for l-rhamnose production. Therefore, the aim of this review is to fill this gap by providing the first comprehensive overview of rhamnose-containing polysaccharides from microalgae, macroalgae, and cyanobacteria, which remain comparatively understudied. This work was conducted within the framework of the ongoing RAh project funded by the French National Research Agency [ANR-23-CE43-0011]. Aquatic organisms such as microalgae and cyanobacteria could provide an attractive alternative, offering the main advantage of avoiding arable land competition while contributing to CO_2_ fixation. Their exploitation could be integrated into intelligent, fully integrated biotechnological processes combining optimized cultivation systems, the hydrolysis of l-rhamnose-containing molecules, and adapted purification technologies.

Variability in polysaccharide composition has been reported within the same species, which can be attributed to differences in cultivation conditions, as demonstrated for *Limnospira platensis* [29]. Analytical bias also contributes to this variability, due to the absence of a universal method for compositional analysis. Polysaccharide characterization requires depolymerization into monosaccharides, usually achieved by acid hydrolysis. However, complete cleavage of glycosidic linkages is difficult; for example, uronic acid linkages require harsh conditions such as concentrated HCl at high temperatures, which simultaneously degrades other monosaccharides into dehydrated compounds related to furfural and lactone derivatives. Conversely, milder conditions, such as hydrolysis with trifluoroacetic acid, preserve monosaccharides but fail to hydrolyze highly stable linkages [30]. These analytical drawbacks may lead to variations in the reported rhamnose content and limit direct comparisons between studies. Indeed, depending on the hydrolysis conditions and the nature of the glycosidic linkages involving rhamnose, the depolymerization procedure may result in incomplete rhamnose release or partial degradation of the released monosaccharides. Such analytical biases may lead to an underestimation of the absolute rhamnose content or to an apparent overestimation of its relative abundance if other monosaccharides are preferentially affected. With an industrial perspective in mind, only polysaccharides with a high rhamnose content were retained in this review. The literature review was conducted primarily using Google Scholar and subscription-based scientific journals accessible through the university library. The search strategy employed combinations of the following keywords: cyanobacteria polysaccharides rhamnose, microalgae polysaccharides rhamnose, and macroalgae polysaccharides rhamnose. Additional relevant publications were identified through the reference lists of articles. Studies were included if they reported the monosaccharide composition of polysaccharides derived from cyanobacteria, microalgae, or macroalgae, with a focus on rhamnose-containing polysaccharides. The aim was to identify the most promising polysaccharide sources for the future industrial production of rhamnose. Therefore, only studies reporting polysaccharide fractions containing at least 20% rhamnose were included in the comparative analysis. References cited for each species thus correspond to cultivation and/or extraction conditions yielding rhamnose-rich polysaccharides rather than representing all compositional data available in the literature for these species. Polysaccharides containing less than 20% rhamnose were excluded, as alternative biological sources would likely be more suitable for industrial rhamnose production. The literature search yielded publications published between 1972 and 2023. The final literature search was conducted in December 2025.

## 2. Sources of Rhamnose-Rich Polysaccharides and Rhamnolipids from Seaweeds, Microalgae, and Bacteria

### 2.1. Polysaccharides from Cyanobacteria

Cyanobacteria, also known as Cyanobacteriota, are a phylum of Gram-negative phototrophic bacteria. They produce O_2_ by oxidizing H_2_O and synthesize organic compounds by reducing CO_2_. As the first organisms to produce and release O_2_, they contributed to the emergence of aerobic life. Cyanobacteria possess photosynthetic membranes, and comparative genomic analyses of protein sequences have confirmed their role as the evolutionary ancestors of chloroplasts. Some species can also fix atmospheric nitrogen, reducing it to ammonia. In filamentous cyanobacteria, specialized cells called heterocysts lose photosystem activity and fix nitrogen, while other cells provide carbohydrates derived from photosynthesis [31]. These carbohydrates can be incorporated into structural polysaccharides. They fulfill multiple roles in cyanobacteria, including adhesion, structural support, protection against abiotic stress, facilitation of gliding motility, and nutrient storage. EPS produced by cyanobacteria display high structural diversity. Glucose is the most frequently reported monosaccharide, although in some strains other sugars predominate. Notably, several cyanobacterial species produce rhamnose-rich polysaccharides (Table 1, Figure 2).

Among the cyanobacterial species reviewed, rhamnose-rich polysaccharides have been reported across six orders (Chroococcales, Gomontiellales, Nodosilineales, Nostocales, Oscillatoriales, and Spirulinales) and eleven families. All these polysaccharides are heteropolysaccharides composed of between 2 and 8 identified neutral monosaccharides. Their compositions are therefore highly heterogeneous. However, when considering the richest rhamnose fractions for each strain in each article, some clear trends emerge. The monosaccharide heatmap (Figure 2) shows the general abundance and recurrence of glucose in rhamnose-rich polysaccharides from cyanobacteria. Indeed, the neutral sugar most frequently associated with rhamnose is glucose (97%), followed by xylose (88%), galactose (81%), mannose (72%), fucose (59%), arabinose (47%), and ribose (22%). Fructose is the least common, occurring in only three of the reviewed polysaccharides. In addition, some polysaccharides contain methyl, acetate, and pyruvate substituents, but sulfate groups are the most prevalent, being present in more than one-third of the reported fractions with the highest rhamnose content, with levels ranging from trace amounts to 20% of the weight. However, the reported frequency of these substituents may be underestimated because their presence is not systematically investigated in the studies considered. Sulfation is noteworthy because it is frequently associated with promising bioactivities, including antiviral [32,33], antioxidant [34], or anticoagulant properties [35]. Uronic acids are present in nearly all cyanobacterial rhamnose-rich polysaccharides. When identified, they are mainly glucuronic acid and galacturonic acid. *Limnospira platensis* polysaccharides have also been reported to contain mannuronic acid and guluronic acid [36]. By contrast, amino sugars have rarely been detected, with only two studies reporting their presence in *Limnospira platensis* [37] and in *Synechocystis* sp. PCC 6714 [38]. The production of anionic EPS may be explained as a response to the high osmotic pressure in saline environments. These strains may have been genetically adapted by producing anionic EPS as a means of protecting themselves from their environment through their high capacity for cation chelation. Moreover, by binding divalent cations, the microorganisms can form a gel that facilitates biofilm formation.

**Table 1:** Sources of rhamnose-rich polysaccharides from Cyanobacteria.

Species	Rha Content (%)	Other Carbohydrates (%)	Composition Details	Type of Polysaccharides	Linkage Information	Reference
*Chroococcales cyanobacterium* LEGE 19970	30 ^b^	Glc (50), Man (4), Xyl (trace), Fuc (trace), Gal (12)		RPS	NA	[39]
*Anabaena spiroides 183 CY BB07* ***Dolichospermum spiroides***	21.9%	Glc (29.3), Man (24.2), Xyl (7.8), GlcA (6.6), Fuc (5.6), Gal (2.0), GalA (1.8), Ara (0.8)		EPS	Enzymatic degradation of EPS by heterotrophic microorganisms	[40]
*Arthrospira platensis* ***Limnospira*** ***platensis***	55.1 ^a^	Glc (8.8), Xyl (10.12), GlcA (7.33), Gal (4.11), GalA (5.57), Fru (6.16), Ara (2.79)		EPS	NA	[41]
*Arthrospira platensis* ***Limnospira*** ***platensis***	49.7 ^a^	Glc (4.3), Man (0.9), Xyl (5.9), GlcA (15.1), Gal (5.8), GalA (16.9), Unknown carbohydrate (1.4)	Sulfates (20% weight)	EPS Fraction PUF2	NA	[35]
*Cyanothece* sp. Viet Nam 01	50.1 ^d^	Glc (17.9), Gal (6.2), Xyl (6.2), Man (3.1), Fuc (4.5), Ara (2.6), Rib (0.8), Unknown carbohydrate (8.4)	Sulfur content (8.4 mol% per total monosaccharide) Uronic acids (23 mol% per total monosaccharide)	EPS	NA	[42]
*Cyanothece* CE4	29.5 ^d^	Gal (0.6), Glc (37.2), Ara (10.3), Xyl (13.6), Fuc (8.9), GalA (Present)	Uronic acids (80.1% of the total carbohydrates) Acetate (0.66% of the total carbohydrates) Pyruvate (0.36% of the total carbohydrates) Sulfates (trace)	EPS	NA	[43]
*Cyanothece* IR20	70.9 ^d^	Gal (0.7), Glc (0.7), Man (16.3), Rib (0.7), Fuc (10.6), GlcA (Present)	Uronic acids (9.8% of the total carbohydrates) Acetate (0.75% of the total carbohydrates) Pyruvate (2.11% of the total carbohydrates) Sulfates (abundant)	EPS		
*Cyanothece* TP5	26.3 ^d^	Glc (24.4), Ara (11.6), Xyl (7.4), Fuc (30.5), GalA (Present)	Uronic acids (40.4% of the total carbohydrates) Pyruvate (1.10% of the total carbohydrates) Sulfates (abundant)	EPS		
*Cyanothece* TP10	23.5 ^d^	Glc (15.0), Man (2.4), Ara (41.1), Fuc (18.1), GlcA (Present), GalA (Present)	Uronic acids (31.3% of the total carbohydrates) Pyruvate (3.86% of the total carbohydrates) Sulfates (present)	EPS		
*Nostoc calcicola* RDU-3	29.67%	Rib (36.03), Xyl (34.13), Glc (4.00), Gal (12.00)	Sulfates (present)	EPS	NA	[44]
*Phormidium* cf. *autumnale* CCALA no. 30	50.2 ^c^	Glc (8.2), Gal (8.4), Xyl (11.1), Man (5.7), Fuc (4.2), Ara (12.2)	Uronic acids (5% weight)	EPS	NA	[45]
*Scytonema ocellatum* CCALA no. 16	42.6 ^c^	Glc (19.8), Gal (6.7), Xyl (12.4), Man (12.6), Fuc (2.1), Ara (3.8)	Uronic acids (3% weight)	EPS	NA	
*Trichormus* sp. CCALA no. 14	37.5 ^c^	Glc (21.7), Gal (4), Xyl (5.1), Man (10.6), Fuc (10.2), Ara (4.9)	Uronic acids (8% weight)	EPS	NA	
*Arthrospira maxima* UTEX LB2342 ***Limnospiramaxima***	20.4 ^b^	Glc (20.0), Gal (5.6), Xyl (36.6), Man (8.2), Fuc (4.3), GlcA (3.5), GalA (1.4)		EPS	NA	[46]
*Arthrospira platensis* ***Limnospira*** ***platensis***	21.0%	Glc (33.1), Gal (7.3), Xyl (3.8)		EXP	NA	[47]
*Arthrospira platensis* SCSIO-44012 (wild type) ***Limnospiraplatensis***	35.41 ^a^	Glc (27.33), Gal (2.37), Xyl (2.79), Man (1.16), Fuc (7.21), Ara (1.11), Rib (6.52), GlcA (0.98), GalA (0.71), ManA (9.49), GulA (4.92)	Sulfates (3.50% dry weight of polysaccharide extracts) Uronic acids (5.93% dry weight of polysaccharide extracts)	EXP	NA	[36]
*Phormidium corium* 674A	23.6 ^d^	Glc (13.4), Gal (11.7), Xyl (27.4), Man (23.9)	Uronic acids (40.7% weight)	RPS	NA	[48]
*Phormidium corium* 442D	22.1 ^d^	Glc (28.5), Gal (11.6), Xyl (15.3), Man (5.9), Fuc (12.7), Ara (3.9)	Uronic acids (11.1% weight) Sulfates (0.7% weight)	RPS	NA	
	25.7 ^d^	Glc (23.8), Gal (16.6), Xyl (13.2), Man (4.4), Fuc (12.6), Ara (3.7)	Uronic acids (10.8% weight) Sulfates (trace)	CPS	NA	
*Phormidium ambiguum* 672B	23.7 ^d^	Glc (19.0), Gal (18.0), Xyl (15.9), Man (6.1), Fuc (10.5), Ara (6.7)	Uronic acids (13.9% weight) Sulfates (trace)	RPS	NA	
	21.5 ^d^	Glc (22.6), Gal (17.6), Xyl (16.5), Man (8.3), Fuc (8.9), Ara (4.5)	Uronic acids (14.0% weight) Sulfates (trace)	CPS	NA	
*Leptolyngbya foveolarum* 671B ***Nodosilineafoveolarum***	21.6 ^d^	Glc (23.6), Gal (18.5), Xyl (15.7), Man (20.5)	Uronic acids (6.4% weight)	RPS	NA	
*Calothrix pulvinata* 7713E	23.3 ^d^	Glc (29.1), Gal (4.2), Xyl (11.5), Man (15.9), Fuc (6.5), Ara (9.4)	Uronic acids (29.2% weight) Sulfates (trace)	CPS	NA	
*Phormidium autumnale* VRUC 164 ***Microcoleus autumnalis***	65.69 ^b^	Glc (3.31), Gal (0.55), Xyl (0.35), Man (2.93), Fuc (0.17), Rib (21.87), GalA (2.59), Fru (2.55)		RPS	NA	[49]
*Microcystis aeruginosa f. aeruginosa* NIES 87	66.7 ^d^	Man (18.8), Glc (14.5)	Uronic acids (5.24 µg/mg dry weight)	EPS	NA	[50]
*Microcystis wesenbergii* NIES 112	0–30.4 ^d^	Xyl (0–1.9), Glc (12.6–100), Gal (0–55.1)	Uronic acids (1.51–5.30 µg/mg dry weight)	CPS extracts	NA	
*Anabaena* sp. *CCC 745*	22.24 ^b^	Glc (32.53), Xyl (32.58), GlcA (11.48), Unknown carbohydrate (1.17)		CPS	Glycosidic linkage analysis using partially methylated alditol acetate derivatization	[51]
*Spirulina* sp.	53.7%	Glc (14.5), Gal (5.6), Xyl (4.4), Man (2.1), Rib (10.0), Unknown carbohydrate (11.1)		EXP	NA	[52]
*Spirulina platensis* ***Limnospira*** ***platensis***	49.4 ^b^	GlcA (9.7), Fuc (8.9), Gal (9.1), Xyl (9.7), Glc (4.1), Man (2.4), GalA (2.0), Ara (1.8), Amino sugar (1.5), N-GlcNAc (0.9), Glycerol (0.4)	Methyl (21.9% of monosaccharides)	EXP fraction immulina	Glycosidic linkage analysis using partially methylated alditol acetate derivatization	[37]
*Spirulina platensis* ***Limnospira*** ***platensis***	44.2 ^b^	Glc (4.7), Gal (2.4), Xyl (0.6), Man (0.8), Fru (33.9), Rib (2.2), GlcA (7.1), GalA (4.0)	Sulfur content (3.24%)	EXP fraction calcium spirulan	NA	[33]
*Spirulina platensis* ***Limnospira*** ***platensis***	89.2%	Xyl (4.8), Other sugars (trace amounts)	Uronic acids (GalA and GlcA) (16.5%) Sulfur content (5.7%) Methyl (Present)	EXP further purified calcium spirulan fraction	Glycosidic linkage analysis using partially methylated alditol acetate derivatization	[53]
*Synechocystis* sp. PCC 6714	4.7–28.7 ^d^	Glc (3.2–59.1), Man (4.0–10.8), Xyl (4.8–13.8), Fuc (3.6–20.7), Gal (6.9–10.3), Ara (0.0–9.3), 3-*O*-methylpentose (1.7–6.5), 4-*O*-methylhexose (0–18.5)	Uronic acids (14.3–20.0% weight of sugars) Sulfates (0–1.2% weight) Methyl (1.7–22.7% of monosaccharides) Osamines (4.8–16.0% weight of sugars) GlcN (62.3–93.3 mol% of osamines) GalN (6.7–37.7 mol% of osamines)	EPS extracts	NA	[38]
*Synechocystis* sp. BASO507	0–92%	Glc (0.98–7), Xyl (0–98), Ara (0–1), GlcA (0.01–1), GalA (0–0.01)		EPS extracts	NA	[54]
*Rhabdoderma* cf. *rubrum* CH	36.6 ^b^	Glc (12.2), Man (12.2), Xyl (4.9), Fuc (2.4), Gal (12.2), Ara (12.2), GlcA (4.9), GalA (2.4)	Uronic acids (6% weight) Sulfates (17% weight)	CPS	NA	[55]

^a^: % weight/weight total sugar; ^b^: % mol/mol total sugar; ^c^: % weight/weight of total neutral sugars; ^d^: % mol/mol of total neutral sugars. EXP: extracted polysaccharides; EPS: exopolysaccharides; RPS: released polysaccharides; CPS: capsular polysaccharides; NA: not available; Rha: rhamnose; Gal: galactose; Glc: glucose; Xyl: xylose; GlcA: glucuronic acid; Man: mannose; Fuc: fucose; Ara: arabinose; GalA: galacturonic acid; ManA: Mannuronic acid; GulA: Guluronic acid; Fru: fructose; Rib: ribose; N-GlcNAc: N-Acetyl-Glucosamine; N-GalNAc: N-Acetyl-Galactosamine; GlcN: Glucosamine; GalN: Galactosamine. Name in bold corresponds to the taxonomically accepted name for the species listed in AlgaeBase; https://www.algaebase.org/ (lastly accessed on 10 September 2026) [56] when the name differs from the original article.

**Figure 2:**
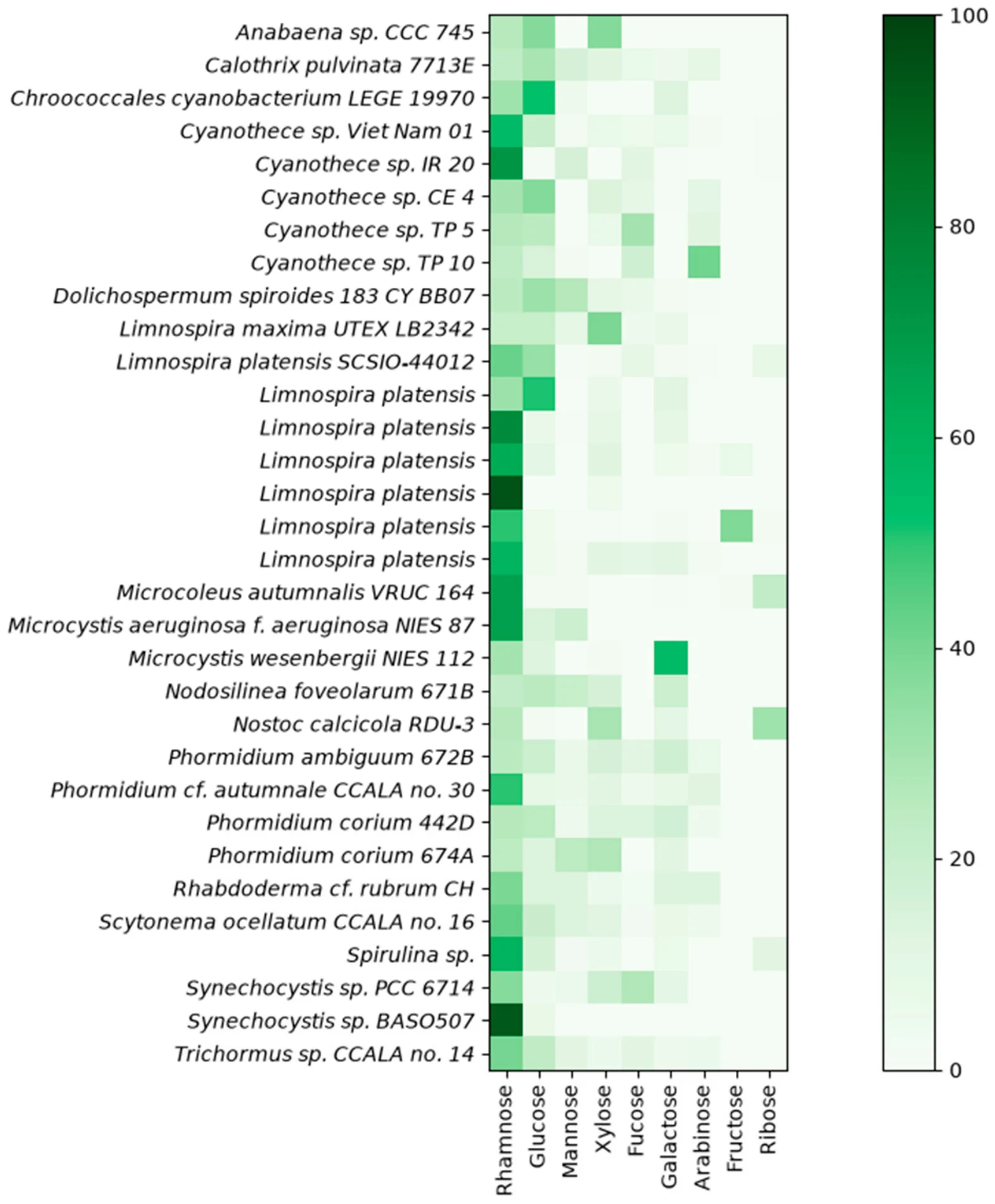
Heatmap of the identified neutral monosaccharide composition of polysaccharides from cyanobacterial species. Each row corresponds to data sourced from the articles listed in Table 1, presented in the original units as reported. For studies including multiple extraction protocols or polysaccharide fractions, the polysaccharide with the highest rhamnose content was considered. Due to variations in methodologies and unit standards, direct comparisons across studies are limited. Shades of green indicate the relative percentage of each monosaccharide.

Among the polysaccharides richest in rhamnose, *Cyanothece IR20* produces an EPS containing 71% rhamnose [43]. Another noteworthy organism is *Microcystis aeruginosa f. aeruginosa*, which reaches 66.7% rhamnose in its EPS, with mannose, glucose, and uronic acids as the only other monosaccharide components [50]. Lee et al. reported up to 89.2% rhamnose in *Limnospira platensis*, although this was observed only in a polysaccharide fraction obtained after successive purification steps [53]. Under salt stress at 0.2 M NaCl, *Synechocystis* sp. BASO507 shifts from producing an EPS without rhamnose to synthesizing one containing 92% rhamnose, while simultaneously increasing the total amount of EPS produced [54].

While considerable diversity of polysaccharide compositions has been described in the literature, glycosidic linkages have been far less explored. Only four studies in Table 1 have studied them. When available, the analyses relied on partially methylated alditol acetate derivatization, which provides information on the position of the glycosidic linkage within each monosaccharide residue but not on the identity of the linkage partner [57]. One study reports the activity of glycosidases from heterotrophic microorganisms [40]. To date, no NMR-based structural study has been reported for the rhamnose-rich cyanobacterial polysaccharides listed in this review.

### 2.2. Seaweed Polysaccharides

Macroalgae, commonly referred to as seaweeds, are multicellular eukaryotic organisms. Among their common characteristics, they exhibit photosynthetic capacity, lack specialized tissues such as roots, and are sessile. They play a key role in the ecosystem, providing food and shelter for aquatic life. The commercial use of macroalgae is expanding, particularly in the Asian food market and as feedstocks for biofuel production [58]. Polysaccharides from seaweeds have numerous industrial applications. Notable examples include carrageenan, a sulfated polygalactan extracted from red seaweeds, which is primarily used in the food industry for its gelling, thickening, and stabilizing properties [59]. Alginate, a polysaccharide composed of l-guluronate and d-mannuronate residues, and extracted from brown algae (*Phaeophyceae*), has applications in biomedicine due to its biocompatibility and gelling capacity [60]. Fucoidan, a sulfated polysaccharide used in the food industry, is mainly composed of fucose residues substituted with sulfate and acetyl groups, along with other monosaccharides, and is extracted from brown seaweeds [61]. Among seaweed polysaccharides, some contain significant levels of rhamnose (Table 2, Figure 3). The rhamnose-rich polysaccharides identified in this review have been reported across five orders and six families. Rhamnose-rich polysaccharides are principally found in polysaccharides from green algae of the Ulvophyceae class, notably the genus *Ulva*, but have also been reported in the brown algal species *Sargassum horneri* and *Undaria pinnatifida*, as well as in the red alga *Grateloupia lithophila*. These polysaccharides include ulvan, a sulfated polysaccharide from the cell walls of green algae, mainly from the order *Ulvales*. Ulvans have been extensively studied [26,62] and contain rhamnose in proportions ranging from 5.0 to 92.2 mol% [62]. Among the most rhamnose-rich ulvans described in this review, Thanh et al. reported a rhamnose content of 85.5 mol% rhamnose among neutral sugars in *Ulva lactuca*, with an ulvan yield of 15.0% based on the dry algal weight. In contrast to cyanobacteria, more information on glycosidic linkages is available, notably through NMR analysis. The polysaccharide structure consists of a major disaccharide →4)-β-d-GlcA-(1→4)-α-l-Rha3S-(1→and other minor disaccharides β-GlcA-(1→2)-α-Xyl and β-GlcA-(1→2)-α-Rha [63]. This structure corresponds to the general ulvan backbone, composed of rhamnose, xylose, glucuronic acid, and iduronic acid linked via α- and β-(1,4) glycosidic linkages. The ulvan backbone is structurally diverse and includes characteristic repeating disaccharide units: aldobiuronic or ulvabiuronic acids, type A and B, respectively, composed of glucuronic acid or iduronic acid linked to rhamnose, and aldobioses or ulvanobioses, type U, composed of xylose linked to rhamnose, with rhamnose generally sulfated at C-3. Type U is less common than types A and B. Minor linkages such as 1,2 and 1,3 linkages, as well as branching, also occur [62].

Among seaweeds, the genus *Monostroma* also produces high levels of rhamnose in its polysaccharides. Species of this genus produce a sulfated rhamnan containing 61.8 to 98.7 mol% rhamnose, with promising anticoagulant, antiviral, and anti-inflammatory activities [25]. The maximum rhamnose content of 98.7% was observed in the fraction PF2 isolated by Li et al., consisting of a sulfated rhamnan from *Monostroma angicava* with a yield of 8.7% (*w*/*w*). PF2 is composed of →3)-α-l-Rha*p*-(1→and →2)-α-l-Rha*p*-(1→residues, with partial branching at C-2 of →3)-α-l-Rha*p*-(1→residues. Sulfate groups are located at C-3 of →2)-α-l-Rha*p*-(1→residues [64]. This corresponds to the general structure of rhamnan sulfate from the *Monostroma* genus, with a backbone of →3)-α-l-Rha*p*-(1→, →2)-α-l-Rha*p*-(1→, and →2,3)-α-l-Rha*p*-(1 →residues. The sulfate groups can be positioned on the rhamnose at the C-2, C-3, and C-4. It can be located at C-1 of the reducing end of →3)-α-l-Rha*p* residue. The backbone also includes →4)-β-d-Glc*p*A-(1→and β-d-Xyl*p*4S-(1→residues. In the branching structure, non-sulfated or mono-sulfated 2- or 3-linked α-l-rhamnose, 4-linked β-d-xylose, and 4- or 6-linked d-glucose residues are present [25].

**Table 2:** Sources of rhamnose-rich polysaccharides extracted from seaweeds.

Species	Rha Content (%)	Other Carbohydrates (%)	Composition Details	Type of Polysaccharides	Linkage Information	Reference
*Enteromorpha compressa* ***Ulva compressa***	7–57 mol% of anhydro sugar	Xyl (15–35), Gal (8–15), Glc (18–50)	Uronic acids (4–14% weight) Sulfates (3–6% weight)	EXP extracts	Glycosidic linkage analysis using partially methylated alditol acetate derivatization, enzyme hydrolysis, and NMR analysis for selected samples	[65]
*Enteromorpha prolifera* ***Ulva prolifera***	32–50 ^b^	GlcA (10–19), Glc (12–42), Xyl (8–20)	Sulfates (16.0–19.1%)	EXP extracts	NA	[66]
*Enteromorpha prolifera* ***Ulva prolifera***	60 ^b^	GlcA (21), Xyl (19)	Uronic acids (14.4%) Sulfates (7.7%)	EXP	Study of enzymatic hydrolysis products, ESI-MS, ESI-CID-MS/MS, and NMR analysis	[67]
*Enteromorpha compressa* ***Ulva compressa***	5–62 (mol% of anhydro sugar)	Ara (0–1), Xyl (15–25), Man (trace–1), Me-Hex (0–8), Gal (0–4), Glc (15–70)	Sulfates (4–13% or not determined) Uronic acids (9–18% weight or not determined) Methyl (0–8% of the monosaccharides)	EXP extracts	Study of enzymatic hydrolysis products for selected sample, MALDI-TOF analysis	[68]
	trace–67 ^d^	Ara (trace–6), Xyl (12–36), Man (0–28), Me-Hex (0–1), Gal (0–7), Glc (3–87)	Sulfates (17% weight or not determined) GlcA (1–18% weight) Methyl (0–1% of the monosaccharides)	Sub-fractions recovered from anion exchange chromatography of the oxalate soluble fraction	Glycosidic linkage analysis using partially methylated alditol acetate derivatization for selected sample	
*Enteromorpha intestinalis* ***Ulva intestinalis***	65.4–67.1 ^d^	Gal (0–3.8), Xyl (14.3–19.2), Glc (11.5–18.6)	Uronic acids (11.4–17.8% weight) Sulfates (8.7–19.9% weight OSO3^−^)	EXP extracts	NA	[69]
*Gayralia oxysperma*	54–88 ^d^	Glc (3–21), Xyl (4–17), Ara (0–10), Man (0–3), Gal (0–6)	Uronic acids (9.7–19.0%) Sulfates (21.2–30.9% SO_3_Na)	EXP fractions	Glycosidic linkage analysis using partially methylated alditol acetate derivatization and NMR analysis for selected samples	[70]
*Gayralia brasiliensis*	61.6–79.7 ^d^	Xyl (8.9–11.5), Gal (1.5–7.8), Glc (7.8–20.2)	Uronic acids (11.4–13.4%) Sulfates (0–36.8% SO_3_Na)	EXP fractions	Glycosidic linkage analysis using partially methylated alditol acetate derivatization and NMR analysis for selected samples	[71]
*Gayralia oxysperma*	76.0 ^d^	Xyl (17.3), Glc (4.4), Gal (2.3)	Uronic acids (17.5%) Sulfates (26% SO_3_Na)	Fraction OX of EXP resulting from the combination of OX-2 to OX-5	Glycosidic linkage analysis using partially methylated alditol acetate derivatization, Smith degradation, and NMR analysis	[72]
*Monostroma latissimum*	72.2 ^a^	Xyl (20.9), Man (0.1), Glc (3.1), Gal (1.1), GlcA (2.6)	Uronic acids (4.9% weight) Sulfates (16.3% weight)	EXP	NA	[73]
*Monostroma nitidum*	0–95.7%	Glc (0–98.6), Xyl (0.8–4.3)	Uronic acids (0–21.8%) Sulfates (1.8–17.7%)	Crude EXP and fractions	NA	[74]
*Monostroma nitidum*	88.83%	GlcA (11.17)	Uronic acids (12.10%) Sulfates (34.80%)	EXP fraction MWS	Glycosidic linkage analysis using partially methylated alditol acetate derivatization and NMR analysis	[75]
*Monostroma angicava*	98.7%	Gal (trace), Xyl (trace)	Sulfates (26.6% weight)	Fraction PF2 of EXP	Glycosidic linkage analysis using partially methylated alditol acetate derivatization and NMR analysis	[64]
*Monostroma angicava*	94.0 ^d^	Xyl (4.5), Glc (1.5)	Sulfates (21.8%) Uronic acids (4.6%)	EXP	NMR analysis	[76]
*Monostroma nitidum*	78.1 ^b^	Man (1.6), GlcA (4.7), Glc (7.8), Gal (1.6), Xyl (6.3)	Sulfates (31% weight)	EXP fraction RS	NMR analysis	[77]
*Monostroma oxyspermum* ***Gayralia oxysperma***	69.32%	Fru (7.80), Xyl (3.01), Gal (2.33), Glc (1.99)	Uronic acids (7.8%) Sulfur content (17.8%)	EXP	NMR analysis	[78]
*Ulva ohnoi*	33.2–53.1 ^b^	Glc (1.3–6.9), Man (0.3–5.3), GlcA (23.6–29.4), IdoA (5.5–10.5), Xyl (5.2–7.3), Fuc (0.2–3.4), Gal (1.2–6.4), Ara (0.0–1.0), Rib (0.6–6.1), GlcN (0–0.9), GalN (0–0.4)	Uronic acids (9.2–23.8% weight) Sulfates (7.1–12.5% weight)	EXP extracts	NMR analysis	[79]
*Ulva lactuca*	85.5 ^d^	Gal (2.6), Xyl (6.0), Man (0.9), Glc (5.1)	Uronic acids (21.5% weight) Sulfates (18.9% weight)	EXP	NMR and ESIMS analysis	[63]
*Ulva pertusa* ***Ulva australis***	60.90%	Xyl (8.50), GlcA (30.50)	Sulfates (present)	EXP fraction F_2_	Glycosidic linkage analysis using partially methylated alditol acetate derivatization and NMR analysis	[80]
*Ulva armoricana* ***Ulva lacinulata***	89.6% weight of the neutral monosaccharides in soluble material	Xyl (9.2), Glc (1.2)	Uronic acids (59 mg/g soluble material) Sulfur (84 mg/g soluble material)	Ulva extract 0625001	NA	[81]
*Ulva armoricana* ***Ulva lacinulata***	26.1–55.6 ^a^	Gal (3.6–6.7), Glc (9.2–48.8), Xyl (2.5–5.4), Uronic acid (8.8–16.5), Other sugars (8.2–23.5)	Uronic acids (8.8–16.5%) Sulfates (14.3–19.1% weight or not determined)	EXP extracts and isolated fraction	NA	[82]
*Ulva lactuca*	36.5 ^b^	Glc (23.8), Ara (19.5), Xyl (5.3), GlcA (14.90)	Sulfates (32.3%)	EXP fraction S1	Study of enzymatic hydrolysis products	[83]
*Ulva lactuca*	34.6–41.4 ^a^	Glc (3.5–16.0), Xyl (15.0–15.1), Man (0.1–0.1), GlcA (28.8–34.9), IdoA (4.9–5.6)	Sulfates (degree of sulfation <0.5–14.3)	EXP extracts	NMR analysis	[84]
*Ulva compressa*	40.7 ^a^	Glc (8.6), Xyl (10.3), Man (0.1), GlcA (32.8), IdoA (7.5)	Sulfates (degree of sulfation 9.3)	EXP	NA	
*Ulva armoricana* ***Ulva lacinulata***	40.8 ^a^	Glc (9.6), Xyl (7.4), Man (0.1), GlcA (26.6), IdoA (15.5)	Sulfates (degree of sulfation 8.7)	Reference ulvan	NMR analysis	
*Ulva lactuca*	51.2–60.8%	Xyl (12.3–14.2), Glc (8.2–20.1), GlcA (16.4–16.8)	Sulfates (12.0–26.8%)	EXP	Smith degradation and NMR analysis	[85]
*Ulva rigida*	46.2 ^d^	Fuc (trace), Ara (4.7), Xyl (38), Gal (trace), Glc (4.2)	Uronic acids (47%) Sulfates (present)	EXP PF-WSE fraction	NA	[86]
*Ulva intestinalis*	30.18–39.13%	Glc (39.05–48.39), Gal (0.0–15.79), Xyl (8.53–11.31), Ara (0.00–5.10)	Sulfates (6.25–24.50%) Uronic acids (4.90–5.95%)	Crude EXP and fractions	Glycosidic linkage analysis using partially methylated alditol acetate derivatization for selected samples	[87]
*Ulva prolifera*	9.29–42.17 ^b^	Man (0.00–1.05), GlcA (2.58–22.77), GalA (0.24–0.91), Glc (24.37–87.22), Gal (0.00–1.90), Xyl (0.00–7.54), Ara (0.00–0.93)	Sulfur content (5.77–16.28%)	EXP extracts	NA	[88]
*Sargassum hornery* ***Sargassum horneri***	7.3–30.6 ^d^	Fuc (69.4–90.1), Gal (0.0–8.1), Xyl (0.0–3.3)	Sulfates (0–16.9% SO_3_Na)	EXP fractions	NMR analysis	[89]
*Undaria pinnitafida* ***Undaria pinnatifida***	40.30–51.44 ^b^	Xyl (17.66–25.34), Gal (10.28–34.48), GlcA (7.56–12.94)	Sulfates (33.99–34.29%) Uronic acids (9.98–12.97%)	EXP fractions	NA	[90]
*Grateloupia lithophila*	79.82–95.88%	Fru (2.21–8.38), Gal (0–3.95), Xyl (1.13–3.31), Glc (0–1.48)	Sulfur content (0.11–6.74%)	Crude and purified EXP	NMR analysis	[91]

^a^: % weight/weight total sugar; ^b^: % mol/mol total sugar; ^d^: % mol/mol of total neutral sugars. EXP: extracted polysaccharides; NA: not available; Rha: rhamnose; Gal: galactose; Glc: glucose; Xyl: xylose; GlcA: glucuronic acid; Man: mannose; Fuc: fucose; Ara: arabinose; GalA: galacturonic acid; Fru: fructose; Rib: ribose; N-GlcNAc: N-Acetyl-Glucosamine; N-GalNAc: N-Acetyl-Galactosamine; GlcN: glucosamine; GalN: galactosamine; IdoA: iduronic acid; NMR: Nuclear Magnetic Resonance; MS: Mass Spectrometry; ESI: Electrospray Ionization; CID: Collision-Induced Dissociation; MALDI-TOF: Matrix-Assisted Laser Desorption/Ionization Time-of-Flight. Name in bold corresponds to the taxonomically accepted name for the species listed in AlgaeBase; https://www.algaebase.org/ (lastly accessed on 10 September 2026) [56] when the name differs from the original article.

**Figure 3:**
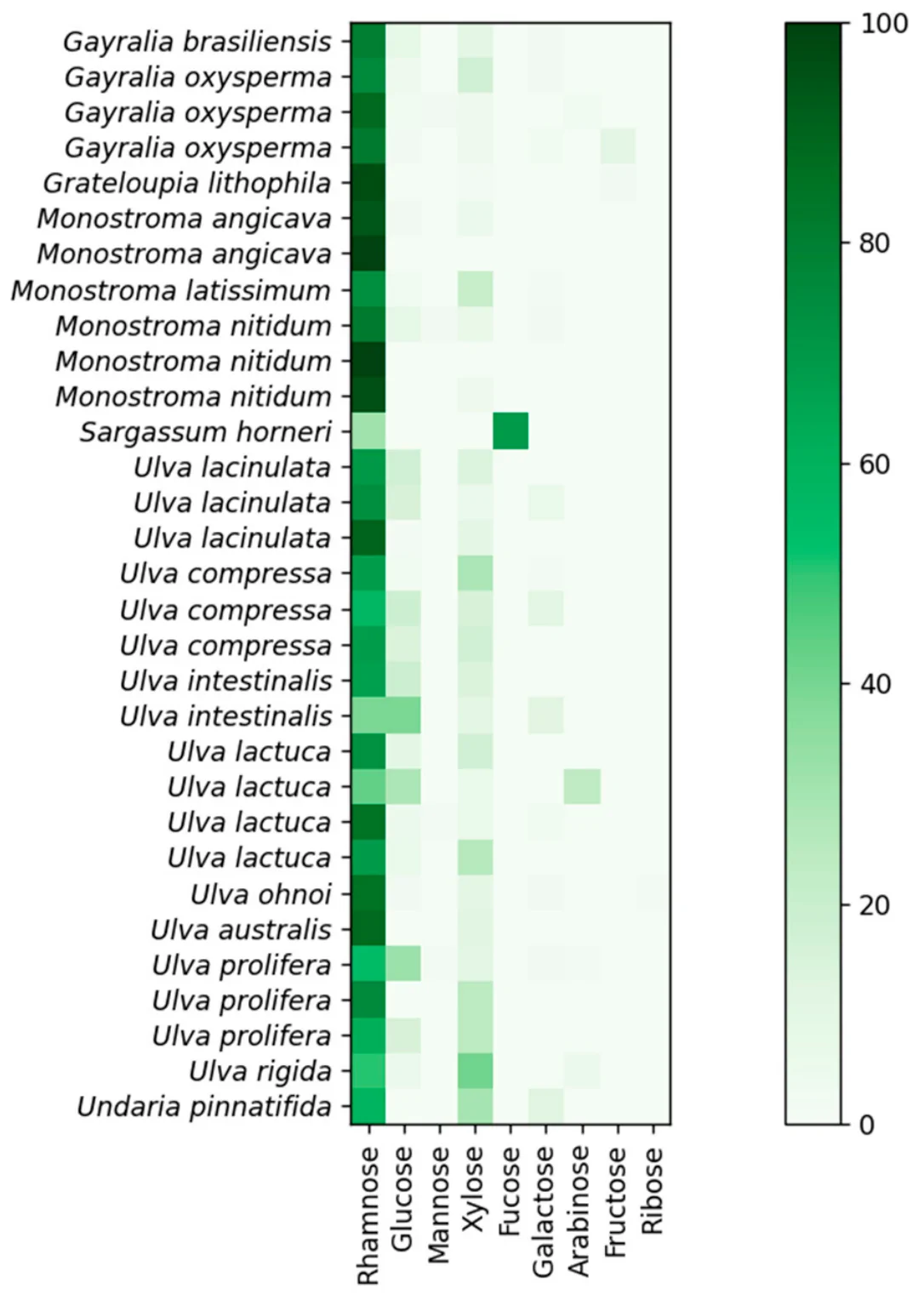
Heatmap of the identified neutral monosaccharide composition of polysaccharides from macroalgae species. Each line corresponds to data sourced from the article listed in Table 2, presented in the original units as reported. For studies including multiple extraction protocols or polysaccharide fractions, the rhamnose-richest polysaccharide was considered. Due to variations in methodologies and unit standards, direct comparison across studies is limited. Shades of green reflect the relative percentage of each monosaccharide.

In the overall context, all the polysaccharides listed here are heteropolysaccharides, and the number of distinct monosaccharides is generally lower than that found in cyanobacteria; this can be partly explained by the focus of the studies on fractions of crude polysaccharides. Considering the rhamnose-richest fraction of each polysaccharide reviewed, the neutral monosaccharides present alongside rhamnose are mainly xylose (present in 94% of cases), glucose (74%) and galactose (48%). The heatmap (Figure 3) highlights the prevalence of xylose and glucose in the composition of these rhamnose-rich polysaccharides. Xylose is frequently the principal neutral monosaccharide (excluding rhamnose) in terms of concentration. Some polysaccharides contain methyl groups but with cyanobacteria the main reported substitution is sulfate. Sulfates are detected in almost all the polysaccharides from macroalgae reviewed here. Likewise, uronic acids are found in 90% of the rhamnose-rich polysaccharides from macroalgae discussed in this review; when identified, they are mainly glucuronic acid residues. However, the genus *Ulva* frequently contains iduronic acid in its polysaccharides.

### 2.3. Microalgal Polysaccharides

Microalgae are unicellular photosynthetic eukaryotic microorganisms. Some species produce polysaccharides with high levels of rhamnose (Table 3, Figure 4). Microalgal polysaccharides are generally heteropolysaccharides often conjugated with non-carbohydrate compounds such as sulfate [92]. Among microalgae, the rhamnose-rich polysaccharides listed in this review belong to fourteen orders and fifteen families. When considering the rhamnose-richest fraction of each polysaccharide, it is noteworthy that all are heteropolysaccharides. *Glossomastix* sp. RCC 3514 exhibits the simplest neutral sugar composition, containing only rhamnose and fucose, which is highly advantageous for downstream purification of rhamnose. Eighty-six percent of the polysaccharides reviewed are composed of five to seven different neutral monosaccharides. The most commonly present monosaccharides are galactose (94%), xylose (86%), glucose (83%), mannose (83%), fucose (69%), and arabinose (56%). Galactose is frequently the dominant monosaccharide (excluding rhamnose) in terms of concentration. Arabinose is much more prevalent in P*lantae* than in *Chromista* (83 vs. 28%). Many species contain uronic acids in their polysaccharides (75%), and when specified, these are either glucuronic or galacturonic acids. A few species also contain hexosamine sugars, including *Auxenochlorella protothecoides*, *Aulacoseira granulata*, and a *Chlorella* sp. The richest rhamnose polysaccharides are produced by *Asterionellopsis socialis* (70%) and *Tetraselmis convolutae* RCC 1564 (61–63%), comparable to the maximum levels observed in cyanobacteria. Regarding non-sugar compounds, most of the articles do not indicate whether the polysaccharide is sulfated. As in cyanobacteria, little additional information is available on the glycosidic linkages of these polysaccharides. However, NMR analyses have been performed on some polysaccharides. For instance, Wan et al. elucidated the structure of a polysaccharide extracted from *Auxenochlorella pyrenoidosa*, which is mainly composed of eight types of residues, including two α-rhamnose units substituted at position 2 [93]. The limited number of studies reporting structural information for polysaccharides from microalgae and cyanobacteria reflects the current analytical challenges associated with polysaccharide sequencing techniques. Cyanobacteria and microalgae polysaccharides are generally heteropolysaccharides which generally lack repeating units. In addition, the absence of a single type of bond as seen in DNA and proteins, generates a great diversity of compounds. Moreover, the limited solubility and high viscosity of aqueous solutions of these macromolecules complicate their analysis by techniques such as NMR. The high structural diversity generates many peaks with relatively low individual signal intensity, making their detection difficult. Moreover, they are distributed over a narrow range of chemical shifts, resulting in spectral overlaps and complicating the spectral interpretation. The structural elucidation of these compounds can only be obtained after following a specific strategy including coupling analytical techniques, chemical modification, and partial depolymerization. The latter allows us to overcome the limitation of NMR analysis by studying oligosaccharide fragments separately. These oligosaccharides can be obtained after uncontrolled or partially controlled methods such as acid hydrolysis or by controlled methods such as carbohydrate-active enzymes when available. However, this requires the purification of the oligosaccharides and a complete analysis of each of them. Chemical modification can provide additional information on the structure. For example, the sulfate frequently present in these polymers may be positioned by comparative analyses of the native and desulphated polysaccharides by techniques such as glycosidic linkage analysis. Mass spectrometry is another major method for determining the monosaccharide sequence. However, this technique is limited by the large number of stereoisomers. It is a key point for cyanobacteria and microalgae polysaccharides which are frequently heteropolysaccharides. Across the main monosaccharides identified there are four different families of stereoisomers which are mainly observed: rhamnose/fucose, galactose/glucose/mannose, ribose/xylose/arabinose, and glucuronic acid/galacturonic acid. Thus, methods for sequencing polysaccharides are not as advanced as DNA sequencing methods. An appropriate analytical strategy is therefore essential to successfully resolve these complex structures. Consequently, the structural elucidation of these polymers is a costly and time-consuming process. This drawback limits the industrial valorization of these compounds. Indeed, the reported biological properties cannot yet be correlated with the structure of these biomolecules. A detailed understanding of the structure–property relationships would make it possible to rationally develop polysaccharides with tailored structures for targeted applications. For example, controlling the position of sulfate groups could be used to modulate their biological activities but the impact of each position on their properties needs to be determined and tools for such specific modifications need to be developed. Thus, developing appropriate analytical tools is a bottleneck for further research and biotechnological valorization of the biopolymers. Intensive research is currently underway to develop future techniques based on concepts such as the use of exohydrolases and gel electrophoresis, scanning tunneling microscopy imaging, or nanopore analysis [94].

### 2.4. Rhamnolipids from Marine Sources

The marine environment contains more than 50% of the global prokaryotic biomass [109]. This diversity provides a source of potential marine bacteria capable of producing rhamnose-rich compounds such as rhamnolipids. Rhamnolipids consist of one or two rhamnose residues linked to a lipid moiety, which grants them biosurfactant properties. The principal source of rhamnolipids is *Pseudomonas aeruginosa*, a pathogenic bacterium. However, some marine bacteria have been identified as rhamnolipid producers and could serve as sustainable alternatives to *Pseudomonas aeruginosa* in the future [110]. For example, some of these marine rhamnolipid-producing strains are listed in Table 4. Among them, the MARISURF project, aimed at identifying novel marine biosurfactants, reported two marine bacterial strains producing rhamnolipids, namely *Marinobacter* sp. and *Pseudomonas mendocina* [111]. However, rhamnolipids have not been detected in cyanobacteria.

Rhamnolipids can be a promising source of rhamnose, and processes for their extraction and purification are already described in a patent. l-rhamnose can be separated from the lipid moiety by hydrolyzing an acidic emulsion of rhamnolipids at temperatures between 100 and 200 °C. After cooling, the aqueous and organic phases are separated, and the pH of the solution is raised by the addition of a basic compound. The precipitate is removed and the solution is concentrated. Purification can subsequently be performed using ion-exchange chromatography. In one of the patent examples, crystals of rhamnose with a purity of 98% have been obtained [118]. Mention should also be made of the enzymatic separation of rhamnose from rhamnolipids. Trummler et al. used naringinase, which has α-l-rhamnosidase activity, to cleave the bond between two l-rhamnose units and to cleave the bond between l-rhamnose units and (*R*, *R*)-3-(3-hydroxydecanoyloxy) decanoic acid. The enzyme preferentially cleaves the bond between two rhamnose units of rhamnolipid. They reached up to 45 g/L mixture of rhamnolipids in a fed-batch process. *Pseudomonas* sp. DSM 2874 could again utilize (R, R)-3-(3-hydroxydecanoyloxy) decanoic acid to produce rhamnolipids. However, when the bacteria were cultivated with 2000 U/L of Naringinase, the enzyme was able to completely cleave the bond between the two rhamnose units under cultivation conditions but monorhamnolipids accumulated in the culture [119]. An alternative method for production of rhamnose from rhamnolipid was described by Linhardt et al. The culture was centrifuged to remove the cells, and the supernatant was adjusted to pH 2.5 with sulfuric acid and incubated overnight at 4 °C. The precipitated rhamnolipids were obtained after centrifugation. This precipitate was resuspended in 1M sulfuric acid at a ratio of 1 g of rhamnolipid/100 mL and heated for 2 h at 100 °C. They obtained a maximum of 46 g/L of rhamnolipids after 8 days in batch fermentation with *Pseudomonas aeruginosa* isolate UI29791, corresponding to 23 g/L of rhamnose. In semicontinuous batch fermentation, they obtained an overall volumetric productivity of 6.4 g rhamnolipid/L/day [21].

In conclusion, a remarkably rich diversity of rhamnose-rich polysaccharides is found across cyanobacteria, microalgae, and macroalgae. These complex structures remain largely under-investigated in unicellular organisms, and current data regarding glycosidic linkages, anomeric configurations, the positioning of substituents such as sulfate groups, and even primary carbohydrate sequences are insufficient. Such detailed characterization is essential for understanding the functional relevance of this structural diversity and for establishing the structure–function relationships that underpin their diverse potential biotechnological applications. Furthermore, these insights would significantly facilitate the development of depolymerization strategies for rhamnose release, particularly the selection of specific enzymes for the selective liberation of rhamnose from heteropolysaccharides, followed by its purification from algal matrices. This knowledge gap results from both the intrinsic complexity of these natural polymers and the lack of polysaccharide sequencing technologies comparable to those established for proteins and nucleic acids. The structural elucidation of these molecules requires a multi-analytical approach, including infrared spectroscopy, colorimetric assays, chromatography, mass spectrometry, and nuclear magnetic resonance, combined with strategies such as partial depolymerization into simpler fragments or specific chemical derivatizations, such as the preparation of partially methylated alditol acetates, to infer key structural features of the native polysaccharides.

## 3. Production of Polysaccharides by Microorganisms

### 3.1. Cultivation of Microalgae and Cyanobacteria

Strains are generally grown in controlled environments where parameters such as pH, temperature, agitation, gas flow and composition, light intensity, and the day/night cycle can be precisely regulated. Among these parameters, the chemical composition of the culture medium is a critical factor for experimental reproducibility. Standardized media have been established for cyanobacteria and microalgae, including f/2 [120,121], Conway medium [122], and Bold’s Basal Medium [123] for microalgae, as well as BG11 [124] and Zarrouk’s medium [125] for cyanobacteria (not exhaustive). These media can also be modified to meet the specific nutritional requirements of the strain of interest. These media are designed for autotrophic cultures, in which carbon is supplied as CO_2_. However, heterotrophic and mixotrophic cultivation strategies are also documented, providing additional carbon sources such as sugars, for example, glucose [126] or organic acids such as acetate [127]. CO_2_ can be supplied through dissolved bicarbonate or aeration, either with ambient air or with enriched gas mixtures containing higher CO_2_ concentrations to enhance photosynthesis [128]. In addition to synthetic media, alternative approaches utilize seawater [129] or wastewater [130] as nutrient sources. The use of wastewater could be an appropriate strategy to reduce costs while simultaneously treating the effluent. Studies have reported the use of agricultural effluents [131], domestic wastewater and industrial wastewater [132] for the culture of photosynthetic microorganisms and the production of value-added compounds.

Cultivation can be conducted in controlled environments ranging from simple aerated bottles, used to provide the necessary elements for photosynthesis and photorespiration, to agar plates, which are typically used for isolating pure strains. For larger volumes, photobioreactors (PBRs) are employed. A PBR is a specific type of bioreactor designed to supply light to photosynthetic organisms. Like conventional bioreactors, they are closed systems that allow precise control of conditions such as temperature, pH, and agitation. PBR designs include bubble columns, airlift, flat-panel, horizontal tubular, and stirred-tank PBRs [133]. In airlift systems, mixing is achieved by gas injection. The reactor consists of a central column where gas is injected, driving the liquid upward, while the surrounding liquid moves downward, creating circulation through the gas–liquid emulsion. Airlift PBRs offer the advantage of reduced shear stress, which benefits shear-sensitive microorganisms. Bubble columns consist of a gas inlet at the bottom and an outlet at the top, enabling continuous aeration. The stirred-tank PBR, most like conventional bioreactors, uses a mechanical stirrer to maintain homogeneity. Because light attenuation increases with cell density, thin-layer PBR designs have been developed, such as horizontal tubular PBRs composed of transparent tubes typically less than 0.1 m in diameter [134], and flat-panel PBRs consisting of thin rectangular panels. These configurations provide a high surface area-to-volume ratio and efficient mass transfer [135]. Alternatively, microalgal cultures can be managed in open-air systems consisting of outdoor tanks exposed to natural conditions, or in semi-enclosed systems where tanks are placed indoors to reduce environmental variability. However, open-air systems are vulnerable to fluctuations in volume due to rainfall or evaporation, which can alter salinity [136]. Generally, the main drawbacks of open-air systems are lower biomass concentrations compared to closed systems and a higher risk of chemical or biological contamination. The advantages of open-air systems are the possibility of larger systems and lower costs. The most common open-air system is the raceway pond, composed of shallow ponds separated by baffles. Circulation is achieved by paddle wheels that drive the flow, ensuring mixing and system homogeneity [137]. Note that microalgae and cyanobacteria studies report cultures in semicontinuous and continuous systems to produce EPS. Each configuration is specific to each microorganism and EPS type in terms of location, production, yield, quality, and structural features [138,139].

Microalgae and cyanobacteria are photosynthetic microorganisms, meaning that autotrophic or mixotrophic cultures require either artificial or natural illumination. Sunlight is a free resource, but its intensity and availability fluctuate diurnally and seasonally. Artificial light provides a constant supply, supporting higher productivity but significantly increasing cultivation costs. It has been estimated that artificial lighting adds approximately $25.3 per kilogram of dry biomass, with a conversion efficiency of 4 to 6% between electrical energy and chemical energy stored in microalgal biomass [140]. Microalgal cultures are generally grown under day/night cycles because photosynthesis involves two phases. In the light phase, chemical energy is produced, while in the dark phase this energy is consumed for carbon fixation [141]. Some organisms grow under continuous light and achieve maximum biomass under this condition, whereas others exhibit photoinhibition and reach peak biomass only under a day/night cycle [142]. The spectral quality of light can also be modulated; the optimal spectral composition depends on the species and the desired metabolite production. For instance, *Galdieria sulphuraria* grows optimally under pure red light, whereas *Porphyridium purpureum* prefers a more balanced spectrum with α_red_:α_green_:α_blue_ of 40:40:20 [143]. Light intensity is another critical parameter that varies by species and applications, with reported values ranging from 62.5 μmol m^−2^ s^−1^ to 2500 μmol m^−2^ s^−1^ [144].

### 3.2. Strategies for Optimizing Polysaccharide Production

Different strategies can improve polysaccharide production by modifying culture parameters to redirect microbial metabolism toward polysaccharide synthesis. A common approach is the application of abiotic stress. Microalgae respond to abiotic stress by altering their metabolic pathways to adapt to environmental changes, which can trigger the production of stress-related metabolites [145]. Accordingly, several studies have explored the enhancement of EPS production under stress caused by heavy metals, light irradiance, temperature, nutrient deficiency, and salinity [146]. Although these conditions generally reduce biomass production, they often result in higher polysaccharide productivity. For this reason, a two-step cultivation strategy is frequently employed, i.e., first using a medium optimized for biomass growth and then switching to a medium promoting polysaccharide accumulation [92]. However, there is no universal method, as responses vary by strain. For example, nitrogen depletion increases polysaccharide production in some cyanobacteria and microalgae, but has no effect in others [147]. Salinity stress is another frequent strategy. Mishra and Jha showed a concomitant increase in EPS production with NaCl concentrations ranging from 0.5 to 5 M in *Dunaliella* s*alina* [148]. Zhou et al. found that in *Auxenochlorella protothecoides*, salinity stress reduced biomass but increased polysaccharide production by 1.64-fold at 10‰ of NaCl [95]. Vo et al. tested seven carbon sources and two salinity levels for a *Chlorella* sp., reaching the highest yield at 3.5% salinity with glycine as the carbon source [149]. Optimization of culture conditions is also effective. Singh and Das investigated temperature, pH, photoperiod, and monochromatic light to enhance production of a rhamnose-rich polysaccharide from *Nostoc calcicola* RDU-3 [44]. Ohki et al. showed that maximum growth rate occurred at 30 °C under 40 μmol photons m^−2^ s^−1^, at 35 °C under 80 and 160 μmol photons m^−2^ s^−1^, and at 40 °C under 80 μmol photons m^−2^ s^−1^, whereas maximum EPS production was achieved at 30 °C under 8 μmol photons m^−2^ s^−1^. This highlights that maximum polysaccharide production does not necessarily coincide with maximum biomass. Interestingly, in this strain, comparable biomass and EPS production were obtained whether nitrogen was provided as NaNO_3_ or under diazotrophic conditions [42]. Metabolic redirection strategies have also been tested. For instance, addition of glyoxylate, an intermediate of the glyoxylate pathway derived from the citric acid cycle, enhanced EPS production in both microalgae [150] and cyanobacteria [151,152]. Furthermore, metabolic engineering offers potential for higher yields and more standardized polysaccharide structures. Yet, this approach requires extensive resources, detailed metabolic route maps and genomic knowledge, and is more challenging in eukaryotes such as microalgae compared with bacterial systems [146]. Optimizing culture conditions to specifically enhance the production of the rhamnose-rich polysaccharide, or to increase its rhamnose content, appears challenging. Such an effort would require an in-depth analysis of the strain genome to identify the metabolic pathway responsible for polysaccharide biosynthesis, as well as the regulatory elements governing this pathway. Although this approach would be highly strain-specific, it could pave the way for genetic modifications aimed at improving rhamnose yields.

Increasing EPS production also introduces rheological challenges, notably due to rising viscosity. Cultures initially behave like water and are easily mixed, but as viscosity increases, problems arise with stirring, homogeneity, and aeration. Downstream processes are likewise affected, with high viscosity complicating separation [21,153]. Filtration systems, for example, are susceptible to clogging under high-viscosity conditions [147]. In bioreactors producing non-Newtonian EPS, shear-thinning behavior occurs. Viscosity then decreases in high-shear zones due to stirring, while remaining higher in low-shear regions. This creates heterogeneity and leads to issues such as limited oxygen transfer, even with increased gas flow, as well as difficulties in pH regulation and culture monitoring. Overall, critical trade-offs between microbial physiology, productivity, and economic feasibility must be carefully balanced during process optimization [153].

Moreover, the scale-up of a future process based on the valorization of microalgal rhamnose-rich polysaccharides represents a major challenge. Indeed, while the development of a greener alternative to current production processes is a key objective, maintaining production costs at a competitive level remains essential to ensure the economic viability of such an approach. As discussed previously, open-air cultivation systems, such as raceway ponds, are considerably less expensive than closed photobioreactors. However, these systems are highly susceptible to environmental fluctuations, including variations in light availability, temperature, evaporation, and weather conditions. Consequently, critical parameters governing EPS production such as light intensity, salinity, and culture temperature are difficult to control and may vary substantially, leading to reduced process robustness and productivity. Another issue is light penetration. Indeed, high cell density is desirable but promotes self-shading. Light is a key substrate in photobioreactors, but it is externally provided and absorbed by the microorganisms, limiting light accessibility to a significant proportion of cells, reducing the growth and productivity of processes in large systems [154]. These different obstacles are critical steps for developing novel green processes based on photosynthetic microorganisms to replace traditional methods, whether to produce rhamnose or other compounds. Nevertheless, photosynthetic microorganisms are already cultivated at industrial scale, particularly *Limnospira platensis*. This species has already been reported to produce rhamnose-rich polysaccharides; notably, its exopolysaccharides (Table 1) and biomass are sold as spirulina. Thus, we can consider the concept of biorefinery where specific strains of *Limnospira platensis* could be cultivated under optimized conditions to promote biomass production and rhamnose-rich polysaccharide expression, to co-produce both rhamnose and spirulina to reduce the cost of rhamnose.

However, despite the substantial increase in global algae production (including both cultivated and wild-collected biomass), which rose from 0.56 million tons per year in 1950 to 35.82 million tons in 2019 according to the Food and Agriculture Organization of the United Nations, microalgae and cyanobacteria account for only a very small proportion of this production. They represent approximately 0.16% of the total output, with 56,456 tons produced, of which 56,208 tons correspond to spirulina alone. Therefore, industrial-scale production of macroalgae is currently far more advanced. Consequently, the development of a low-cost rhamnose production process appears, at present, to be more feasible using macroalgae as a biomass source rather than microalgae or cyanobacteria. Among the different species of seaweed, the *Ulva* production represents 2356 tons per year and could be a source of rhamnose-rich polysaccharides (Table 2). However, green seaweeds (excluding microalgae) only represent 0.09% of the total production and this is far less than red seaweeds (51.48%) and brown seaweeds (47.65%) [155]. Among them, certain species produce rhamnose-rich polysaccharides (Table 2). It is noteworthy that macroalgae cultivation remains highly dependent on biotic and abiotic conditions, which can lead to seasonal variations and fluctuations in biomass production, such as for photosynthetic microorganisms in open air systems. Thus, bioreactor-based cultivation systems with controlled and consistent production throughout the year while minimizing the risk of environmental contamination are still a relevant approach for producing high-value-added compounds.

In conclusion, the productivity of the strains represents a genuine bottleneck. Significant challenges arise from the generally low yields observed for EPS, as well as from the viscosity that develops when their concentrations increase, particularly during concentration steps in downstream processing. Moreover, based on current knowledge, no universal method exists to intensify EPS production in microalgal or cyanobacterial strains. Consequently, for the purpose of producing rhamnose from rhamnose-rich polysaccharides derived from microalgae and cyanobacteria, only extensive, strain-specific optimization and experimental work can identify the ideal conditions required to achieve maximal productivity. In contrast, genetically modifying cyanobacteria to increase productivity and/or enhance the rhamnose content of their polysaccharides, while maintaining strain viability, would require research efforts of prohibitive complexity, necessitating an in-depth understanding of the metabolic pathways leading to the synthesis of these heteropolysaccharides, whose structures are particularly complex and still insufficiently studied.

## 4. Extraction and Purification of Polysaccharides

The extraction of a rhamnose-rich polysaccharide from biomass, whether derived from macroalgae or microorganisms, avoids the issues associated with viscosity that arise during EPS production. However, polysaccharides obtained through extraction are generally more contaminated by organic compounds than EPS, thereby increasing the burden of downstream purification steps. Extraction of polysaccharides from biomass has been well described [156,157,158], but it generally requires multiple steps. The standard method is hot-water extraction, typically using volumes of water several dozen times greater than the biomass. Variants include acid- or alkali-assisted methods, still using water as the base solvent. Yet these processes are characterized by low efficiency, high temperature requirements, and long extraction times. The overall design of rhamnose production needs to be considered from an environmental sustainability perspective. Thus, the use of green technologies becomes a priority for the extraction of rhamnose-rich polysaccharides. Thus, even though water is the greenest solvent available, greener extraction techniques are explored. To improve efficiency, novel methods have been developed, including physical treatments such as microwaves, ultrasound, pressurized liquids, or pulsed electric fields, as well as enzymatic approaches. The principle is to facilitate cell disruption. Optimization can also involve the fluid used for extraction, for instance by using subcritical water or supercritical fluids. These methods are considered greener for the extraction of polysaccharides [159,160].

Extraction may require pretreatment steps such as centrifugation to separate microorganisms from their medium, drying to remove water, and grinding for multicellular organisms. Biomass can also be suspended in absolute ethanol to extract pigments prior to polysaccharide recovery. Following extraction, purification is performed in successive steps. Centrifugation or filtration is typically the first step to remove insoluble compounds and cellular debris. The supernatant can then be processed to eliminate contaminants. Ethanol precipitation (70–80%, 4 °C) is the most common initial step for recovering polysaccharides from aqueous extracts, although precipitation efficiency depends on structural diversity and should be optimized for the target product [161]. Acetone can be used as an alternative [156]. For microalgae and cyanobacteria, dialysis and filtration may be preferable, since ethanol often co-precipitates salts with polysaccharides [147]. Depending on the type of contamination, different strategies are used. Lipids can be removed using ethanol with petroleum ether [156] or n-hexane [162]. Proteins, being hydrophilic like polysaccharides, represent a frequent contaminant. Several deproteinization methods exist, including the Sevag method, salt precipitation, trichloroacetic acid, functional adsorbents, proteases, and trichlorotrifluoroethane [163,164]. The Sevag method employs a mixture of chloroform and n-butanol for denaturing proteins. Finally, dialysis is used to remove salt and small compounds, and the purified product is dried using techniques such as spray-drying, freeze-drying, or oven-drying to obtain a dry powder.

A universal protocol for polysaccharide extraction does not exist. Indeed, the specific characteristics of the cell walls of different organisms, polysaccharide properties such as solubility or resistance to degradation, and trade-offs between purity, yield, and cost do not allow the development of a generic extraction protocol. Given the diversity of polysaccharide structures and the wide range of organisms, it appears that a single extraction protocol, both optimized and selective for rhamnose-rich polysaccharides, is not feasible. However, specific examples of rhamnose-rich polysaccharides can serve as a basis for formulating targeted recommendations. To begin with ulvan, an acidic and anionic polysaccharide, Kidgell et al. compiled several studies and reported the following points on its extraction. The solubility of ulvan depends on intermolecular interactions mediated by divalent cations such as Ca^2+^. These interactions are pH dependent. Ulvans are more soluble in solutions where the pH < the pKa of uronic acids and sulfate esters because, below the pKa, they lose their negative charge. Their conformation shifts from bead-like structures to a more dispersed state, enhancing solubility. This property is useful for selective extraction, since glucuronan and xyloglucan from *Ulva* are more soluble in alkaline solutions. In addition, proteins are least soluble at their isoelectric point; for instance, the soluble proteins from *Ulva* have an isoelectric point of 2.25. Based on the criteria of high extraction yield, low degradation, and high selectivity, they proposed optimal conditions for aqueous ulvan extraction as 80–90 °C, pH 2–4.5, and a duration of 1–3 h [62]. These conditions are consistent with the findings of Glasson et al. for *Ulva ohnoi*, using multiple response optimization, who determined optimal extraction at a pH of 2.2–4.0, 61.3–90.0 °C, and 55.0–90.0 min [165]. Pretreatment can further enhance extraction. Reducing the salt content of the biomass with warm water decreases ulvan aggregation and causes osmotic shock, increasing exposure of cell wall components. Chelators such as sodium oxalate, which remove divalent cations, improve extraction efficiency, especially at pH 4.5. However, HCl extraction provides higher selectivity than sodium oxalate, resulting in better ulvan purity [79]. Monostroma, another genus of green macroalgae known for producing rhamnose-rich polysaccharides, synthesizes an anionic polysaccharide. Extraction is generally performed with hot water at 65–100 °C for 2–6 h, with a biomass-to-water ratio of 1:10 to 1:50, yielding 15.0 to 28.6%. Multiple or reflux extractions improve yield [25]. Extraction can also be physically assisted. Tsubaki et al. achieved a 51.3% yield using microwave-assisted extraction at 1 kW, with a biomass-to-water ratio of 1:20, an extraction temperature of 140 °C, and a duration of 10 min [166]. Rhamnan sulfate can be purified by anion-exchange resin column chromatography thanks to the sulfate groups which bind to the column before being eluted by increasing the sodium chloride concentration [167]. EPS, by contrast, are simply recovered by centrifugation or filtration from microalgae and cyanobacteria cultures before purification steps. However, high viscosity could lead to difficulties in isolating the polysaccharide. Strategies such as diluting the medium can help with separation. The resulting pellet of biomass can be incubated, for example, with 0.1 M sulfuric acid at 95 °C for 1 h and centrifuged again to recover capsular polysaccharides (CPS) [49].

The choice of acid conditions and the desalting steps for ulvan, for example, is based on the structure of the polysaccharides. However, specific strategies employed for rhamnose-rich polysaccharide extractions are not based on rhamnose properties but on the presence of ionic groups (sulfate, uronic acid) in these polysaccharides. This choice appears consistent given that other neutral monosaccharides exhibit a chemical structure very similar to that of rhamnose, particularly fucose, which is a stereoisomer of rhamnose. Under these conditions, it becomes highly unlikely to develop an extraction method that is truly specific to rhamnose-rich polysaccharides. Thus, polysaccharide extraction can also result in mixtures of polysaccharides differing in molecular weight and composition. Final purification can therefore be performed to obtain more homogeneous products, particularly when targeting rhamnose-rich polysaccharides. Some approaches rely on solubility differences since polysaccharides of different molecular weights and shapes can display different solubilities. Graded precipitation exploits this by gradually increasing the concentration of alcohols or ketones (e.g., ethanol) or neutral salts (e.g., NaCl, KCl, or (NH_4_)_2_SO_4_) [164]. At each step, polysaccharide precipitates are separated by centrifugation, and the process is repeated on the supernatant to recover additional fractions. Polysaccharide precipitation can also be achieved through coordination with metal ions or quaternary ammonium salts. The latter is particularly interesting because precipitation is selective for acidic polysaccharides and neutral high-molecular-weight polysaccharides [164].

The storage polysaccharides in cyanobacteria, macroalgae and microalgae are glucans. These neutral polysaccharides can be separated from anionic polysaccharides by precipitation. Indeed, anionic polymers that have been extracted can be selectively precipitated by a pyridinium salt such as cetylpyridinium chloride, or by a quaternary ammonium salt such as hexadecyltrimethylammonium bromide. This process is based on ionic interactions [168]. However, chromatography methods are the most widely used techniques. Different types of columns are used for separating heterogeneous polysaccharides including cellulose columns, anion exchange columns, gel-filtration columns, and affinity columns [164]. With gel-filtration columns, polysaccharides are separated according to their size as they pass through a column packed with porous beads. Molecules that are too large to enter the pores are eluted first because they are not retained, whereas smaller polysaccharides elute later since they can diffuse into the pores, increasing the path they must travel. The technique separates molecules based on their hydrodynamic volume rather than their exact mass, so a branched polysaccharide may elute before a linear polysaccharide of the same mass. The different columns differ in the matrices used and the size of the pores. They include Sephadex (dextran), Sepharose, Bio-gel, Superose (agarose), TSKgel (silica) and the copolymers Superdex (dextran-agarose) and Sephacryl (allyldextran/N,N′-methylenebisacrylamide). Column selection is primarily determined by the pore size, which must be matched to the dimensions of the macromolecule. This technique can be an appropriate choice for rhamnose-rich polysaccharides, provided that their size differs significantly from that of the contaminating polysaccharides.

Based on the molecular mass, methods such as ultracentrifugation or ultrafiltration could be alternatives. However, ultrafiltration generally faces issues of membrane fouling and low yields over long processing times, whereas ultracentrifugation is typically used only for small quantities due to the required equipment. Neither method appears to be well suited for scale-up [164]. Ion-exchange columns appear to be a promising option for the purification of rhamnose-rich polysaccharides derived from algae and cyanobacteria. Indeed, polysaccharides from photosynthetic organisms are frequently sulfated and/or contain uronic acids as detailed previously. Thus, rhamnose from polysaccharides from *Monostroma* and *Ulva* is sulfated. Moreover, ulvan contains high levels of uronic acids. This makes ion-exchange chromatography an attractive option to separate rhamnose-rich polysaccharides from contaminants such as glucan. In this context, anion-exchange columns are the best suited. It should be noted that anion-exchange chromatography retains polysaccharides through ionic interactions as well as adsorption phenomena, which enables the purification of mixtures containing both neutral and acidic polysaccharides, or exclusively neutral ones. Generally, at pH 6.0, acidic polysaccharides remain in columns whereas neutral polysaccharides are not retained. A solution with a different ionic strength allows elution. Such columns notably include DEAE-Cellulose and DEAE-Sepharose/Sephadex which combine ion-exchange capacity and a molecular-sieving effect [164]. Diethylaminoethyl (DEAE) is a positively charged group attached to the matrix. Neutral polysaccharides can also be separated on anion exchangers using borate anion exchanger columns. At pH > 12, the hydroxyl groups of sugars begin to ionize very slightly, but this charge is negligible and non-selective. There is no significant difference in retention between the isomers. Borate forms specific anionic complexes with monosaccharides, creating a negative charge that varies according to the sugar structure for anionic selectivity. Indeed, borate can form reversible covalent linkages with the cis-diols present in monosaccharides. This method could be useful for separating neutral polysaccharides with similar sizes but different compositions.

As previously discussed, the design of rhamnose production should prioritize eco-friendly approaches. Thus, the different techniques presented should be selected carefully to design the most environmentally responsible process possible, prioritizing those with a low environmental footprint while replacing, wherever possible, the most polluting and toxic ones. Although the extraction step is generally performed using water, subsequent purification and precipitation steps commonly rely on organic solvents. Thus, the elimination of proteins using protocols such as the Sevag method using chloroform and n-butanol should be avoided in favor of novel approaches such as deep eutectic solvents [169] (green solvent) or freeze–thaw treatment [170] (green physical method) as examples. Deep eutectic and natural deep eutectic solvents are obtained from the combination of two or more components that have lower relative melting points in the mixture than individual components due to hydrogen bonds between the donor and acceptor components in a certain molar ratio. They are promising sustainable alternatives for extraction and separation of bioactive molecules because they have low melting points, high solubility and extraction ability, low toxicity, recyclability, non-volatility, and low cost [159,160,171,172]. Other green solvents include water, supercritical fluids, surfactants, ionic liquids, switchable solvents, and biobased solvents. Ionic liquids are salts with low melting points; they are frequently liquid at room temperature, but toxicity has been observed for some of them. Switchable solvents can be converted to hydrophilic and hydrophobic states by varying temperature, pH, CO_2_, light, or magnetic properties. Thus, they can be simply separated and recovered from the solvent system to be recycled and reused while reducing energy consumption [160].

Currently, there is no method for the specific purification of rhamnose-rich polysaccharides from polysaccharide mixtures. However, specific rhamnose-recognizing lectins exist in human keratinocytes and fibroblasts [11,173]. l-rhamnose-binding lectins also exist in other organisms and have already been isolated and characterized, for example, from teleost fish eggs [174] or eggs of steelhead trout [175]. The use of lectins with specific affinity for rhamnose could be envisioned as a strategy to purify rhamnose-rich polysaccharides or even free rhamnose. Indeed, lectins could be immobilized on a matrix to create lectin-affinity chromatography columns for polysaccharides. Such an approach would rely on the selective binding properties of these lectins to capture and isolate rhamnose structures from complex mixtures. However, to date, no studies have reported the application of rhamnose-binding lectins for this purpose, and the feasibility of this method remains unexplored. Further investigation will be required to assess its practical potential and to develop robust purification protocols based on this concept. However, this method has already been employed for the separation of glycoproteins with the well-known concanavalin A, a lectin which is specific for mannose and glucose residues [176]. The major advantage of affinity chromatography is its efficiency in purifying low-abundance polysaccharides; a single round of affinity chromatography can achieve concentration factors ranging from several hundred to several thousand [164,177].

## 5. Strategies for Efficient Rhamnose Liberation from Polysaccharides

The first step in obtaining pure monosaccharides, particularly rhamnose, is the depolymerization of polysaccharides. This can be achieved by different methods applied separately or in combination, including radical depolymerization, acid hydrolysis, acid methanolysis, enzymatic treatment, or physically assisted hydrolysis such as microwaves [178]. Physical methods also exist to reduce polysaccharide size and decrease viscosity, such as high-pressure homogenization [179] or microwave treatment, and these could represent interesting pretreatments. However, they do not allow the reduction of polysaccharides to the monosaccharide scale. Chemical methods are the simplest and lowest-cost methods possible. However, methanolysis is a powerful method for acid polysaccharides but it implies chemical modifications of monosaccharides by methylation. Consequently, additional steps will be needed to eliminate the methyl groups. Radical-mediated depolymerization tends to over-degrade monosaccharides, oxidize them, and generate reactive by-products. Although this approach efficiently fragments polysaccharides, the free radicals involved cleave both intra- and intermolecular bonds, yielding oligosaccharides with newly formed end groups. As a result, radical depolymerization is poorly suited for the selective release of native monosaccharides such as rhamnose [180,181]. Generally, polysaccharides are depolymerized into monosaccharides by hydrolysis. However, the instability of released monosaccharides, the variable resistance of glycosidic linkages, and the structural complexity of polysaccharides prevent the development of a universal method [178]. Acid hydrolysis is the most common approach, followed by neutralization at the end. The principal acids used are sulfuric acid, trifluoroacetic acid (TFA), and hydrochloric acid. Parameters such as acid concentration, reaction time, and temperature must be optimized to achieve complete degradation of all the *O*-glycosidic bonds. Note that excessive parameters can degrade monosaccharides into non-sugar derivatives such as 2-furfural for pentoses or 5-hydroxymethyl-2-furfural for hexoses [178]. Wang et al. tested the stability of 10 different monosaccharide standards under 2 M TFA at 110 °C for up to 6 h. Under these conditions the most stable monosaccharides were amino sugars, then neutral sugars, whereas uronic acids were the least stable. Rhamnose has a loss ratio between glucose and galactose. After 2 h, 15.4% of rhamnose was degraded and 31.7% after 6 h [182]. Hydrolysis conditions of glycosidic linkages have been reported to be quantitative under 0.01 M H_2_SO_4_, 100 °C, 1 h for ketoses, which are the least stable monosaccharides, 2 M H_2_SO_4_, 100 °C, 2 h or 4 M TFA, 100 °C, 4 h for aldoses, 4 M TFA or HCl, 100 °C, 4 h for hexosamines and 2 M H_2_SO_4_, 100 °C, 2 h for uronic acids [183]. However, although uronic acids are less stable toward acid hydrolysis, the uronic linkage is more stable under acidic hydrolysis conditions. Indeed, the carboxyl group of a uronic acid provides electrostatic stabilization to the glycosidic bond by withdrawing electron density from the anomeric carbon, making its acid-catalyzed hydrolysis slower and more energetically demanding. However, the same protonated group weakens the uronic acid itself by promoting decarboxylation and degradation of the uronic monomer under strong acidic conditions. Acidic hydrolysis will be a useful method for polysaccharides like rhamnan from Monostroma. Moreover, sulfate is frequently present in polysaccharides from aquatic photosynthetic resources, and acid hydrolysis can eliminate it. However, this method has limitations and drawbacks. Indeed, this approach requires large volumes of mineral acids, which poses a significant challenge for eco-friendly process development and downstream processing due to the generation of salt by-products that must be removed. Alternatively, organic acids are a possibility. Furthermore, because acid hydrolysis is uncontrolled, all constituent monosaccharides are released, which necessitates a subsequent purification step. Finally, the uronic linkages will be difficult to break without over-degrading rhamnose. Reduction of the uronic acids can be envisaged; methods such as carbodiimide coupled with NaBH_4_ are described in the literature [184]. Moreover, as described above, many polysaccharides from seaweeds, microalgae and cyanobacteria contain uronic acids. For instance, Garna et al. tested several methods for hydrolyzing the neutral sugar chains of pectin, which include rhamnose. They showed that release rates differ among carbohydrates, with rhamnose requiring longer hydrolysis due to its strong linkage with galacturonic acid in the main backbone units of pectin. Among the acids tested, TFA caused less sugar degradation, particularly for rhamnose, than H_2_SO_4_ or HCl. Combining chemical and enzymatic hydrolysis achieved complete hydrolysis without sugar degradation [185]. Quemener et al. studied ulvan hydrolysis. A chemical method with 2M TFA at 120 °C for 3 h failed to completely depolymerize ulvan, as ulvanobiouronic acid (β-d-GlcA-(1,4)-l-Rha) resisted treatment. Meanwhile, 75% of xylose was degraded under these conditions. To overcome this, they combined chemical hydrolysis with β-d-glucuronidase, which enabled complete ulvan hydrolysis without xylose degradation [186]. Thus, acid hydrolysis alone does not always allow complete depolymerization of the polysaccharide while preventing monosaccharide degradation. These limitations highlight the value of using complementary strategies, such as combined approaches involving enzymes, which offer more specific and milder alternatives. Enzymes are natural biomolecules produced by all organisms. Among them, glycoside hydrolases can hydrolyze glycosidic bonds between two or more carbohydrates, or between a carbohydrate and a non-carbohydrate moiety. Polysaccharide lyases can cleave uronic acid-containing polysaccharide chains via a β-elimination mechanism to generate an unsaturated hexenuronic acid residue and a new reducing end [187]. In nature, polysaccharide degradation occurs spontaneously through the activity of living organisms [188]. For example, the fungus *Trichoderma reesei* produces highly efficient enzymes for cellulose degradation, i.e., five β-1,4-endoglucanases, two exoglucanases (cellobiohydrolases), and two β-glucosidases [189]. This natural degradation can be reproduced in the laboratory by using enzyme mixtures. For instance, Bauer et al. cloned 74 genes encoding polysaccharide-degrading enzymes from three fungi to analyze plant cell wall degradation [190]. Meng et al. used a carbohydrase mix to depolymerize non-starch polysaccharides from wheat, soybean meal, canola meal, and peas [191]. In the case of rhamnose, the selection of enzymes capable of lysing glycosidic linkages should lead to the production of l-rhamnose in free form and oligosaccharides. However, this strategy requires, first, the elucidation of the polysaccharide structure and, second, the selection of endo-hydrolases and endo-lyases capable of cleaving the polysaccharide at the rhamnose position and exo-hydrolases to liberate the rhamnose residue. Moreover, sulfates are common, and rhamnose is sulfated in rhamnan and ulvan. So, sulfatases will also be needed or desulfation steps such as solvolysis of pyridium salt. Various enzymatic activities acting on rhamnose have been described. The enzymes that specifically cleave terminal l-Rhamnose are α-l-rhamnosidases (E.C. 3.2.1.40). Different catalytic activities are reported: α-1,2-rhamnosidase, α-1,3-rhamnosidase, α-1,4-rhamnosidase and α-1,6-rhamnosidase. Their optimal pH varies from 2.0 to 11.0, and their optimal temperature ranges from 4 to 95 °C [192,193]. Several studies have reported an inhibitory effect by l-rhamnose, glucose, citric acid, and several metal ions. They are notably distributed in the glycoside hydrolase (GH) family GH78 and GH106. These enzymes have been identified in plants, animals, yeasts, fungi and bacteria [192]. GH106 enzymes are inverting glycosidases, acting by anomeric configuration inversion [194], as is also the case for GH78 enzymes [195,196] releasing β-l-rhamnose, but it is spontaneously converted to the α-form by mutarotation [196]. Note that GH106 enzymes are also Ca^2+^ dependent [197]. It is noteworthy that enzymes within these glycoside hydrolase families exhibit broad substrate diversity. They specifically hydrolyze the non-reducing end of α-l-rhamnose [193]. These families include enzymes capable of cleaving a wide range of glycosidic linkages in polysaccharides. For example, the GH78 family includes an α-l-rhamnosidase that cleaves the α-l-Rha*p*-(1→3)-d-Glc*p* of gellan [198], whereas the GH106 family contains an enzyme capable of cleaving the α-l-Rha*p*-(1→2)-l-Ara*p* linkage present in rhamnogalacturonan II [197]. However, many α-l-rhamnosidases preferentially target flavonoid glycosides, such as rutin, naringin, and hesperidin, among numerous other substrates [192,193]. Consequently, not all members of these families are suitable for polysaccharide depolymerization. Other GH families contain enzymes able to cleave rhamnose linkage. The GH13 family includes also α-l-rhamnosidases [193]. The GH39 family includes an endo-rhamnosidase capable of cleaving α-l-Rha*p*3S-(1→4)-β-d-Xyl*p* in ulvan. GH90 contains a lipopolysaccharide endo-a-1,3-l-Rhamnosidase cleaving α-l-Rha*p*-(1→3)-β-d-Gal*p*. Endo-rhamnosidases are of significant interest to reduce the size of the polysaccharide to increase depolymerization efficiency but it implies that the enzyme recognizes the polysaccharide as a substrate. There are also rhamnogalacturonan-degrading enzymes, GH28 includes rhamnogalacturonan I endo-a-1,2-galacturonidase (E.C. 3.2.1.171) and rhamnogalacturonan I exo-a-1,2-galacturonidase (E.C. 3.2.1.173) capable of cleaving α-d-Gal*p*A-(1→2)-α-l-Rha*p*. In contrast, polysaccharide lyase (PL) families PL11 and PL26 contain enzymes that act on the α-l-Rhap-(1→4)-α-d-Gal*p*A linkage of rhamnogalacturonan. Rhamnogalacturonan is an interesting example structure because galacturonic acid is one of the two main uronic acids identified previously in the polysaccharides. Thus, these enzymes coupled with rhamnogalacturonan α-l-rhamnohydrolases from GH78 could in theory depolymerize rhamnogalacturonan structures. However, this approach needs the production of a variety of recombinant enzymes and moreover, there is a lack of characterized rhamnosidases. CAZy, the Carbohydrate-Active enZYmes database; http://www.cazy.org/ (lastly accessed on 10 September 2026); only indexes 12 rhamnosidases characterized in GH106 and 49 in GH78 while these families contain respectively 2947 and 14,564 enzymes at the time of writing [187]. Thus, in the current state of knowledge, it does not seem relevant to look for enzymes one by one. Fortunately, an alternative exists to find enzymes capable of depolymerizing polysaccharides. This approach is based on in silico study of Polysaccharide Utilization Loci (PUL) that are clusters of genes organized around a susCD gene pair principally in the Bacteroidetes phylum. PULs code for a pool of enzymes capable of depolymerizing a polysaccharide [199]. The first PUL was identified in *Bacteroides thetaiotaomicron* and was an operon encoding enzymes involved in starch degradation where SusC and SusD act together for starch import. For example, Salinas and French used a PUL to depolymerize ulvan. Thus, they identified and expressed 5 enzymes from *Formosa agariphila* [200]. The PUL approach has also been successful for identifying ulvan-degrading enzymes in *Alteromonas* sp. [201]. Databases exist to facilitate the identification of PULs, such as PULDB; https://www.cazy.org/PULDB/ (lastly accessed on 10 September 2026); where searches can be performed with list of CAZy families as entry [199]. Moreover, the existence of bacteria with a PUL capable of depolymerizing the polysaccharide of interest avoids the need to elucidate the complete structure of the polysaccharide for the selection of enzymes. Since enzymes are generally expensive, the best strategy will be to immobilize them for reuse. Note that alternatively, enzymatic hydrolysis of rhamnose-rich biomass can be carried out directly by microorganisms. Kuivanen and Richard engineered *Aspergillus niger* to not consume rhamnose while retaining the ability to release rhamnose by hydrolyzing substrates such as rhamnogalacturonan, pectin, and citrus peel [24]. It should nevertheless be emphasized that, if selective cleavage of the linkages involving rhamnose in the polysaccharide of interest is possible, it would allow the release of rhamnose together with oligosaccharides, thereby facilitating its subsequent purification relative to complete depolymerization. However, this strategy implies a structural architecture with rhamnose in a position facilitating its selective release, characterized enzymes capable of this selective cleavage, and a deep knowledge of the structure. Thus, it will be necessary to identify the activity of the different enzymes in the PUL to eliminate enzymes that do not contribute to the selective release of rhamnose. Complete depolymerization has the advantage of enabling the valorization of co-products such as fucose, another rare sugar that could be produced in parallel with certain polysaccharides, such as those produced by *Glossomastix* species.

Considering the various possible approaches for polysaccharide depolymerization aimed at releasing rhamnose, and considering the available information on algal polysaccharides, the most suitable strategy is likely a combination of acid hydrolysis and enzymatic treatment. Chemical hydrolysis is clearly the least expensive option; however, it does not efficiently cleave uronic linkages. Therefore, in cases where rhamnose is bound to a uronic sugar, the use of enzymes appears to be the most effective compromise, as it prevents rhamnose degradation while maximizing its release. In other situations, namely when rhamnose is linked to a neutral sugar, acid hydrolysis remains the most efficient and cost-effective method. It should also be noted that rhamnose may carry sulfate groups in certain sulfated polysaccharides, such as those found in *Monostroma* and *Ulva*. Nevertheless, the acidic conditions required for complete depolymerization generally led to the cleavage of sulfate groups, making the use of sulfatases or desulfation steps unnecessary. Although the use of enzymes remains more expensive than acid hydrolysis, it nevertheless represents the greener approach, as it limits the use of mineral acids and facilitates purification during downstream processing by avoiding the introduction of additional and substantial amounts of salts.

## 6. Methods for Isolating Rhamnose from Mono- and Oligosaccharide Mixtures

Hydrolysis of heteropolysaccharides releases a mixture of monosaccharides. Therefore, efficient separation is required to achieve high-purity monosaccharide fractions. However, monosaccharides released by hydrolysis are difficult to isolate because of their high structural similarity; several of them are stereoisomers (e.g., rhamnose/fucose, glucose/galactose, arabinose/xylose/ribose) with physicochemical properties lying within very narrow ranges, which further complicates their separation. Efficient purification of rhamnose is therefore critical for its valorization from algal biomass. However, to address this challenge, several techniques have been developed, mainly chromatographic methods, including partition chromatography [202], high-performance liquid chromatography [203], hydrophilic interaction liquid chromatography [204], centrifugal partition chromatography [205], ion-exchange resins [22,23,206], and ion exclusion chromatography [207]. Strongly acidic cation-exchange resins have also been employed in continuous chromatography systems based on simulated moving bed technology [208]. Methods based on nanofiltration also exist [209].

These separation methods rely on differences in the physicochemical properties of monosaccharides, some of which are summarized in Table 5 for the main monosaccharides found in algal polysaccharides. Rhamnose and fucose share a LogP value of −2.1, which is the lowest value among the common monosaccharides found in algal polysaccharides. Due to the absence of a hydroxyl group at the C-6 position, rhamnose is slightly less hydrophilic than other hexoses, although it remains highly water-soluble with a reported water solubility of 521.5 g/L at 40 °C [210].

Neutral monosaccharides such as rhamnose have pKa values around 12, meaning that they can only be retained on anion-exchange columns under strongly basic conditions, typically using 0.1 M sodium hydroxide. This approach is effective for separating rhamnose from uronic acids, which are abundant in algal matrices (e.g., glucuronic and galacturonic acids). However, the method is less selective for neutral sugars. Another strategy involves borate-complex anion-exchange chromatography, in which borate forms reversible covalent complexes with cis-diols, imparting a negative charge to neutral sugars. This enables their separation using borate buffer gradients. This method can also separate monosaccharides and disaccharides in case of intentionally incomplete depolymerization. An advantage of this method is its high tolerance for salts and organic contaminants, making it attractive for algal hydrolysates [211]. However, borate gradients complicate downstream processing and raise environmental concerns. Monosaccharides can also be purified by ligand-exchange conversion. In this method, metals such as sodium, calcium, or lead act as counterions. Sugars form complexes with metal ions through their hydroxyl groups. Water is used as the mobile phase. Using this method, monosaccharides can also be separated from disaccharides, including disaccharides containing monosaccharides other than rhamnose, which may arise from partial depolymerization. Partition methods with materials such as aminopropyl bonded to silica polymer support, with acetonitrile and water as the mobile phase, can also be used to separate monosaccharides from oligosaccharides. Centrifugal partition chromatography (CPC) is based on liquid–liquid partition, in which the stationary phase is retained by the hydrostatic force generated by the centrifugal field in the rotor [212]. This technique enables very high yields because there is no irreversible adsorption due to the absence of a solid support, and it allows for higher flow rates, leading to reduced elution volumes for strongly retained substrates. Ward et al. purified rhamnose from a mixture of arabinose, galactose and galacturonic acid obtained from pectin hydrolysate using ethanol:DMSO:aqueous ammonium sulfate (300 g L^−1^) (0.8:0.1:1.8, *v*:*v*:*v*) achieving a purity of rhamnose >90% [205]. Finally, as previously discussed for polysaccharide purification, affinity chromatography with rhamnose-binding lectins is a promising but underexplored alternative method for the specific purification of rhamnose. Indeed, rhamnose-binding lectins exhibit strong and specific recognition of l-rhamnose. Their use in affinity chromatography could enable the highly selective purification of rhamnose from complex hydrolysates, including those rich in structurally similar deoxyhexoses such as fucose. However, no commercial affinity columns based on rhamnose-binding lectins are currently available, and the development of such materials would require further investigation to characterize and select suitable rhamnose-binding lectins.

## 7. Complete Process for Rhamnose Production from Polysaccharides

Some examples of rhamnose production have been described in the literature. Rhamnose production processes have been developed from several sources, such as gum arabic, a polymer composed of d-galactose, l-arabinose, l-rhamnose, and d-glucuronic acid [213]. A patent describes a process in which this polymer is hydrolyzed using a mineral acid, after which the solution is neutralized, concentrated, and finally precipitated using an organic solvent. The aqueous phase is then purified by chromatography on a strong cationic ion-exchange resin, resulting in a rhamnose purity of 96 to 98%. An additional adsorption and separation step using activated carbon increases the purity to 99.5%. However, approximately 50% of the rhamnose released during the hydrolysis is lost during the precipitation step. Additional steps to treat the precipitate increase the overall yield to 93% of the rhamnose present in gum arabic [23].

Serrat et al. used the *Klebsiella* sp. 1-714 strain to produce a polysaccharide composed of rhamnose, galactose, and glucuronic acid. After chemical hydrolysis of the polysaccharide, *Candida rugosa* was used to remove galactose, a neutral sugar. This enabled the complete removal of galactose. However, inhibitory compounds were present in the solution. Therefore, a prior ultrafiltration or dilution was required. Activated carbon was also tested, but a loss of more than 60% of the rhamnose was observed. The purification protocol used an ion-exchange resin to separate rhamnose from glucuronic acid under batch or continuous annular chromatography conditions. A rhamnose solution free of other sugars was obtained. Water was used as the eluent, with a 2M NaCl solution used as the displacement eluent. Crystallization was performed using ethanol [206].

For algae belonging to the *Monostromaceae* and *Ulvales*, methods are already available for the isolation of l-rhamnose. A patent describes a process beginning with the extraction of sulfated rhamnan using a polar solvent, preferably water, at temperatures ranging from 90 to 160 °C. The polymer is then hydrolyzed to release l-rhamnose, followed by purification steps including desalting, decolorization, ion exchange, and crystallization to obtain purified l-rhamnose. An additional fermentation step using yeast unable to metabolize rhamnose can be performed to improve the purity and yield of l-rhamnose. Among the examples described in the patent, one embodiment involves the treatment of 12 kg of *Monostroma* to produce 1.22 kg of rhamnose monohydrate crystals with a purity of 98.8% [214].

Dumraliya et al. reported the production of rhamnose from ulvan extracted from *Ulva fasciata*. Their process involved the use of a solid acid catalyst to depolymerize ulvan while simultaneously limiting the formation of degradation products. This was achieved using carbon-embedded sulfonated polymers in an aqueous solution containing solubilized ulvan. Following depolymerization, the mixture was deacidified by passing it through two columns packed with a microporous anion-exchange resin, which removed sulfate, uronic acids, and chloride ions. The purified solution was then concentrated and crystallized by cooling, using commercial rhamnose hydrate as a seed, resulting in a purity of up to 85%. The demineralization, concentration, and crystallization of the ulvan hydrolysate yielded a 60.2% yield of transparent crystals in 2–3 days. The carbon-embedded sulfonated polymers facilitated adsorption-assisted hydrolysis of glycosidic and sulfonyl ester bonds while suppressing side reactions leading to the formation of furanic compounds. According to the authors, the process appears economically viable, with a rhamnose selling price of INR 6000, corresponding to approximately USD 63 when considering an ulvan production cost of INR 150 and an overall yield of 1 kg of l-rhamnose hydrate crystal from 20 kg ulvan (semi-wet/air dried) [215]. As discussed previously, this is higher than the average price of rhamnose in 2025, which was USD 46/kg.

The sequence of unit operations required to produce rhamnose from polysaccharides can be summarized as follows. First, the polysaccharides are subjected to hydrolysis. This can be followed by a clarification step involving an organic solvent to precipitate residual macromolecules, such as proteins and non-hydrolyzed polysaccharide fractions. Rhamnose is subsequently separated by chromatography, typically using an ion-exchange resin. The rhamnose-enriched fraction is recovered and concentrated. Finally, purified solid rhamnose can be obtained by crystallization, vacuum evaporation, or lyophilization.

Based on all the information gathered, we propose the following recommendations to produce rhamnose from rhamnose-rich polysaccharides derived from algae or cyanobacteria, as summarized in Figure 5. First, the polysaccharides can be extracted either by centrifugation in the case of EPS or by hot aqueous extraction under acidic conditions, in the presence of ion chelators or other desalting steps. These conditions weaken the cell walls and facilitate extraction. Other physical methods, such as microwave-assisted extraction, may also be employed. In this context, osmotic shock may additionally help disrupt aggregates that can form due to the negative charges carried by uronic acids, while acidic pH also contributes to aggregate dissociation, even though charged polysaccharides are theoretically more soluble in water. Moreover, the isoelectric point of macroalgal proteins is around 3–4, which reduces their solubility; for the soluble proteins from ulvan, it is closer to 2.25. A preliminary depigmentation step may also be required. Once sugar-rich solutions are obtained (after centrifugation or aqueous extraction), successive purification steps can be implemented to remove salts, proteins, or other compounds that may interfere with downstream processes. Polysaccharide recovery is finalized by ethanol precipitation followed by a drying step. The extraction may result in a mixture of polysaccharides. A final concentration and purification step can be carried out using ion-exchange columns or affinity columns based on rhamnose-specific lectins, although the latter still require further development. However, it appears more economically viable to go directly to the depolymerization step of the polysaccharide mixture due to the cost of chromatography, as well as the general low solubility and high viscosity of polysaccharides. When rhamnose is substituted with uronic acid, we recommend the use of specific enzymes selected through PUL analysis. In other cases, namely when rhamnose is linked to neutral monosaccharides, acid hydrolysis remains the simplest method to implement and can efficiently depolymerize rhamnose-containing polymers. Finally, rhamnose can be purified using various established chromatographic techniques, such as ion-exchange chromatography, and CPC or through more innovative approaches involving affinity columns functionalized with rhamnose-specific lectins, although the latter still require further technological development. The most used technique appears to be ion-exchange chromatography. However, CPC appears to be a more promising technology for the industrial-scale purification of l-rhamnose because of its potential for greater cost-effectiveness. Its loading capacity is much higher, and the absence of a solid stationary phase helps reduce costs. Solid-phase columns are expensive and may exhibit decreasing performance over repeated cycles, eventually requiring replacement. They can also become clogged or fouled. CPC, on the other hand, uses solvents in biphasic mixtures. The stationary phase is retained by centrifugal force. These solvents are less expensive and can be recycled, making centrifugal partition chromatography a potentially greener method for producing rhamnose. The last step consists of crystallizing rhamnose from the rhamnose-rich solution by adding rhamnose crystals as seed.

## 8. Conclusions

l-rhamnose has diverse applications in the agro-industry, cosmetics, and pharmaceutical sectors. The growing market therefore creates a demand for new sources of l-rhamnose. Microalgae, seaweeds, and cyanobacteria are highly biodiverse sources of polysaccharides, many of which contain significant amounts of l-rhamnose. In this review, polysaccharides from 25 orders and 32 families are described. The structures of these biopolymers remain poorly understood due to their intrinsic complexity and the lack of sequencing technologies for polysaccharides. Thus, further research is needed to develop novel technologies for polysaccharide sequencing. Elucidating these complex structures is necessary to understand both the functional relevance of such extensive biological diversity and the structure–function relationships governing these polymers, thereby enabling the rational design of polysaccharides with targeted biotechnological properties.

Some polysaccharides, such as rhamnan sulfates from the genus *Monostroma*, already have established processes for l-rhamnose production. However, the vast unexplored biodiversity of aquatic organisms may lead to the discovery of new l-rhamnose-producing organisms. Among the identified candidates, species such as *Cyanothece* IR20, *Microcystis aeruginosa f. aeruginosa*, *Asterionellopsis socialis*, and *Tetraselmis convolutae* RCC 1564 should be considered because of the relatively high rhamnose content of their polysaccharides. However, *Limnospira platensis* may be the most promising candidate. Indeed, this species is already cultivated on an industrial scale to produce spirulina. Thus, rhamnose could be co-produced by valorizing both biomass and exopolysaccharides within a biorefinery framework. This approach could improve the economic competitiveness of l-rhamnose production while taking advantage of an existing biomass valorization market. Such a co-product valorization strategy may be essential, as optimizing culture conditions for microorganisms producing rhamnose-rich polysaccharides, although necessary, may be insufficient on their own to achieve economic competitiveness. Microalgae- and cyanobacteria-based processes still face significant challenges for industrial-scale production because current cultivation systems involve a trade-off between productivity and cost. Considering current world production capacities, macroalgae appear to be the most promising feedstock for sustainable and cost-effective rhamnose production. Further improvements in cultivation technologies and the valorization of low-cost substrates such as wastewater will be required to lower the cost of cultivating photosynthetic microorganisms. In addition, other challenges remain in developing environmentally friendly and economically viable processes. Greener depolymerization processes based on PUL-derived enzyme cocktails and enzyme immobilization could reduce reliance on corrosive reagents. The resulting sugar mixtures are typically separated by anion-exchange chromatography to obtain pure l-rhamnose. However, other methods such as CPC should be considered, given their performance potential and greener profile, resulting from the use of a readily recyclable stationary phase. Innovative methods, such as affinity chromatography using rhamnose-binding lectins, could also provide a solution for separating complex matrices containing multiple monosaccharides, including compounds with structures closely related to rhamnose. Such techniques may enable a several-hundred- to thousand-fold enrichment of rhamnose in a single pass through the column, thereby substantially reducing solvent consumption.

In conclusion, this review highlights the rich biodiversity of aquatic environments and the challenges associated with developing new processes to obtain pure l-rhamnose from aquatic resources. Smart strategies combined with environmentally friendly downstream processing will be essential to establish aquatic photosynthetic organisms as competitive and sustainable alternatives to traditional plant-derived sources of l-rhamnose production.

## Figures and Tables

**Figure 1:**
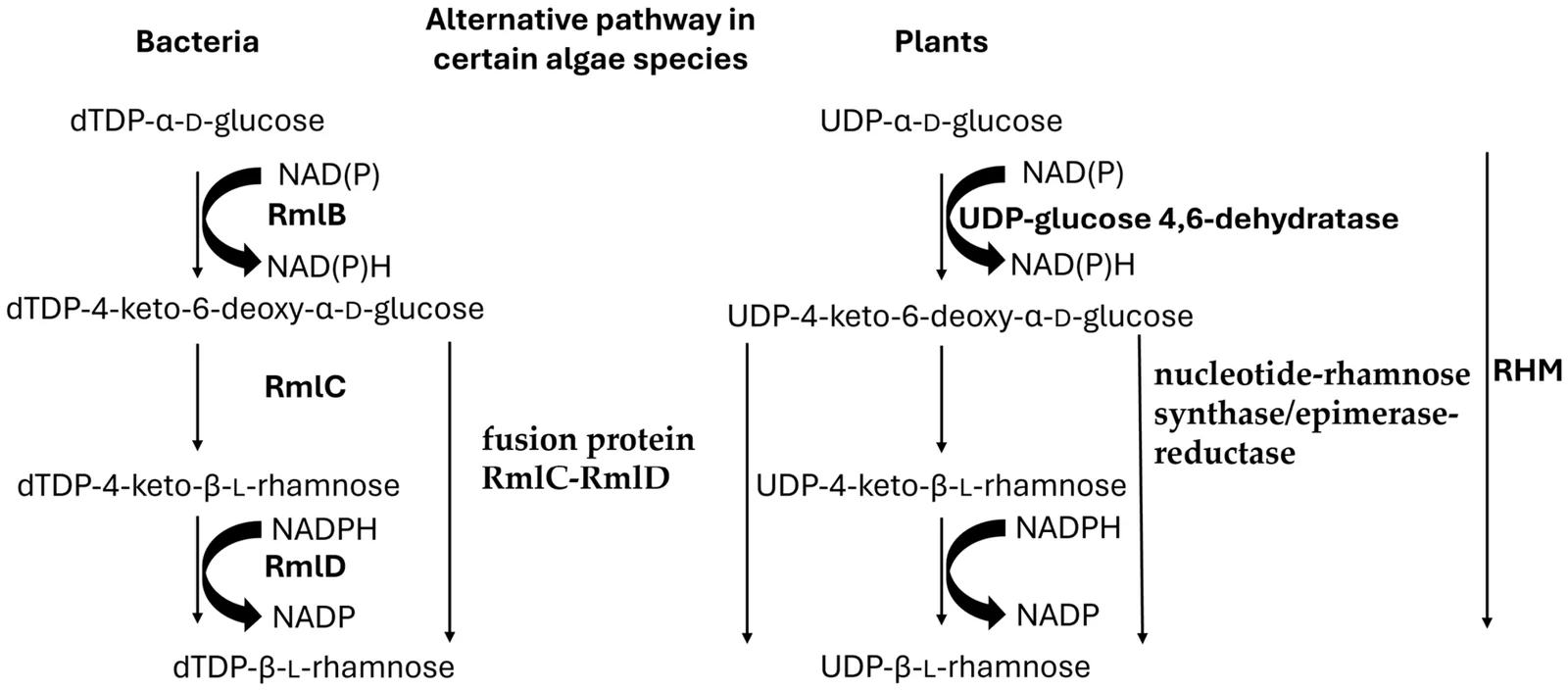
Metabolic pathway of l-rhamnose in bacteria and plants. In bacteria, three different enzymes (RmlB, RmlC and RmlD) catalyze the dehydration, epimerization, and reduction of dTDP-α-d-glucose to produce dTDP-β-l-rhamnose. In plants, the substrate is UDP-α-d-glucose, and the three steps are carried out either by RHM or by UDP-glucose 4,6-dehydratase and nucleotide-rhamnose synthase/epimerase-reductase, leading to the production of UDP-β-l-rhamnose. An alternative pathway involves a fusion protein of RmlC and RmlD in certain algal species, notably within the Haptophyta and Gymnodiniaceae families of microalgae. RHM: rhamnose synthase, UDP: uridine diphosphate, dTDP: deoxythymidine diphosphate, NAD: nicotinamide adenine dinucleotide, NADP: nicotinamide adenine dinucleotide phosphate.

**Figure 4:**
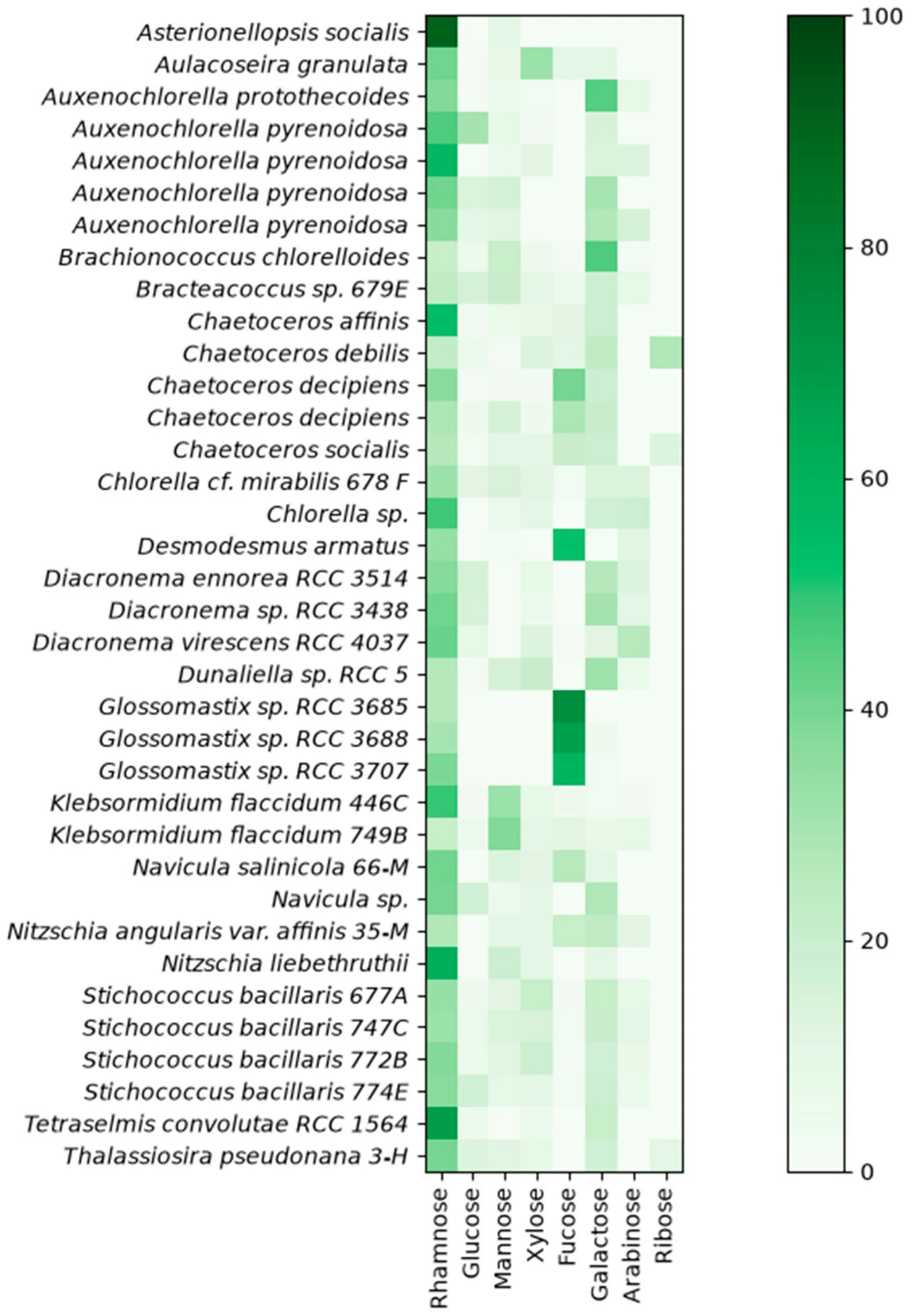
Heatmap of the identified neutral monosaccharide composition of polysaccharides from microalgae species. Each line corresponds to data sourced from the article listed in Table 3, presented in the original units as reported. For studies including multiple extraction protocols or polysaccharide fractions, the rhamnose-richest polysaccharide was considered. In cases where rhamnose levels were identical, or when several measurements were reported for the same fraction, the mean value was retained. Due to variations in methodologies and unit standards, direct comparison across studies is limited. Shades of green reflect the relative percentage of each monosaccharide.

**Figure 5:**
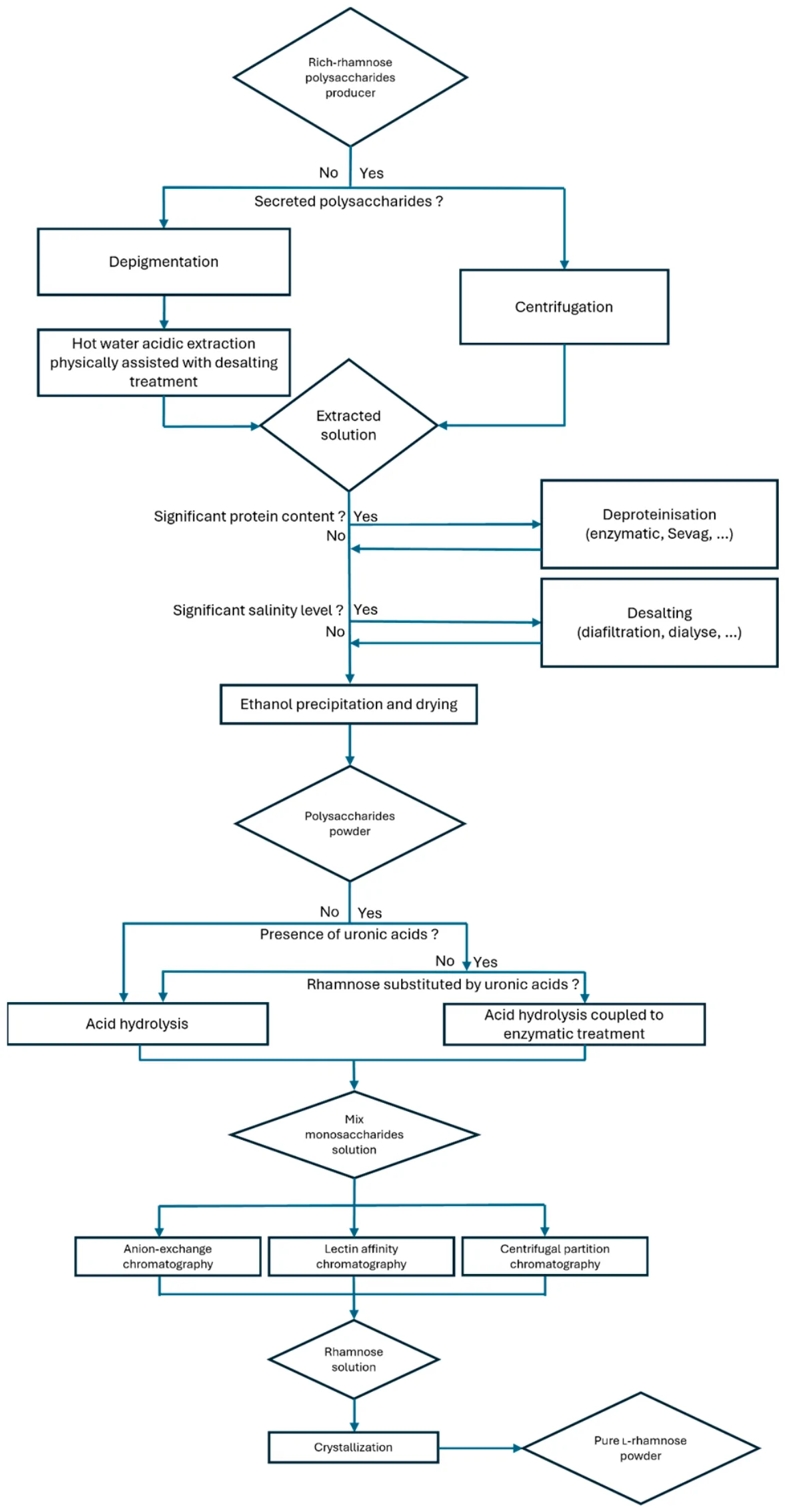
Diagram of the proposed process to produce pure l-rhamnose from rich-rhamnose polysaccharides producers.

**Table 3:** Sources of rhamnose-rich polysaccharides from microalgae.

Species	Rha Content (%)	Other Carbohydrates (%)	Composition Details	Type of Polysaccharides	Linkage Information	Reference
*Auxenochlorella protothecoides*	4.12–35.29 ^b^	Glc (0.70–3.81), Gal (42.41–84.08), Xyl (1.43–2.68), Man (2.03–4.62), Ara (1.84–7.76), GlcN (0–1.68), GalN (0–0.23), GlcA (0.79–6.54)		EPS fractions	NMR analysis	[95]
*Aulacoseira granulata*	31.3%	Xyl (24.5), GlcA (13.2), Fuc (7.8), Gal (7.0), GalA (6.2), Man (5.4), N-GlcNAc (3.2), N-GalNAc (0.7), Glc (0.7)		EPS	Glycosidic linkage analysis using partially methylated alditol acetate derivatization	[96]
*Chaetoceros affinis*	39%	Fuc (35), Gal (26)		EPS	NA	[97]
*Chaetoceros decipiens*	34–36%	Fuc (32–40), Xyl (2–5), Man (2–7), Gal (17–19), Glc (1–5)		EPS extracts	NA	
*Chaetoceros affinis*	25–52%	Fuc (7–11), Rib (0–15), Xyl (7–7), Man (6–11), Gal (18–22), Glc (2–3), 4 Unknown carbohydrates		EXP extracts	NA	
*Chaetoceros debilis*	22–22%	Fuc (8–11), Rib (23–31), Xyl (10–15), Man (0–2), Gal (23–23), Glc (4–6)		EXP extracts	NA	
*Chaetoceros decipiens*	25–27%	Fuc (4–5), Rib (4–10), Xyl (2–10), Man (24–32), Gal (7–10), Glc (0–6), Unknown X_1_ (12–22)		EXP extracts	NA	
*Chaetoceros socialis*	10–23%	Fuc (10–18), Rib (11–20), Xyl (8–12), Man (9–15), Gal (16–19), Glc (3–7), Unknown X_2_ (7–12)		EXP extracts	NA	
*Chlorella* sp.	25.5 ^b^	GlcN (23.4), GalN (17.8), Unidentified aminosugar (5.8), Ara (10.3), Gal (8.9), Xyl (5.5), Man (2.7)		EPS	ESI-MS/MS and NMR analysis	[98]
*Dictyosphaerium chlorelloides* ***Brachionococcus chlorelloides***	2.2–21.0% weight of total sugar including uronic acids	Fuc (0–1.3), Ara (0.7–11.2), Xyl (0.7–6.0), Man (2.6–22.0), Gal (30.5–79.5), Glc (1.4–39.1)	Uronic acids (0–1.7% weight of total sugar or not determined) Methyl (8.6–22.1% of monosaccharides)	Native EPS, insoluble part, and fractions of EPS	Glycosidic linkage analysis using partially methylated alditol acetate derivatization and NMR analysis for selected samples	[99]
*Nitzschia frustulum* var. *perpusilla* ***Nitzschialiebethruthii***	24–43%	Fuc (trace–6), Rib (0–trace), Ara (0–trace), Unknown X (1–14), Unknown Y (3–20), Xyl (trace–7), Man (13–40), Glc (0–trace), Gal (6–23), GlcA (Undetermined amounts)	Sulfates (8.9–9.3% of carbohydrate content or not determined) Methoxyl (0.1–0.2% of carbohydrate content or not determined)	EPS extracts	NA	[100]
*Nitzschia angularis* var. *affinis* 35-M	20%	Fuc (16), Ara (8), Unknown X (4), Xyl (7), Man (7), Unknown Z (21), Gal (17), Glc (trace), GlcA (Undetermined amounts)		EPS	NA	
*Cyclotella nana* 3-H ***Thalassiosira pseudonana***	33%	Fuc (trace), Rib (8), Unknown X (4), Xyl (7), Man (10), Unknown Z (13), Gal (14), Glc (11), GlcA (Undetermined amounts)		EPS	NA	
*Navicula incerta* Grunow 66-M ***Navicula salinicola***	33%	Fuc (20), Ara (trace), Unknown X (7), Unknown Y (3), Xyl (9), Man (10), Unknown Z (10), Gal (8), Glc (trace), GlcA (Undetermined amounts)		EPS	NA	
*Asterionella socialis* ***Asterionellopsis socialis***	70%	Fuc (trace), Unknown X (8), Xyl (trace), Man (7), Unknown Z (15), Gal (trace), Glc (trace), GlcA (Undetermined amounts)		EPS	NA	
*Diacronema* sp. *RCC 3514* ***Diacronema ennorea***	33 ^b^ GC/MS analyses / 37 ^b^ HPAEC-PAD analyses	Glc (15), Gal (26), Xyl (5), GalA (0), GlcA (4), Ara (17) / Glc (16), Gal (23), Xyl (10), GalA (trace), GlcA (4), Ara (8)		EPS	NA	[101]
*Glossomastix* sp*. RCC 3688*	28 ^b^ GC/MS analyses / 20 ^b^ HPAEC-PAD analyses	Gal (4), Fuc (54), GalA (5), GlcA (9) / Gal (2), Fuc (57), GalA (11), GlcA (10)		EPS	NA	
*Glossomastix* sp*. RCC 3685*	20 ^b^ GC/MS analyses / 19 ^b^ HPAEC-PAD analyses	Fuc (56), GalA (14), GlcA (10) / Fuc (57), GalA (13), GlcA (8)		EPS	NA	
*Glossomastix* sp*. RCC 3707*	31 ^b^ GC/MS analyses / 27 ^b^ HPAEC-PAD analyses	Gal (2), Glc (0), Fuc (40), GalA (21), GlcA (6), Xyl (0) / Gal (3), Glc (trace), Fuc (47), GalA (15), GlcA (7), Xyl (trace)		EPS	NA	
*Pavlova* sp. RCC 4037 ***Diacronema virescens***	46 ^b^ GC/MS analyses / 36 ^b^ HPAEC-PAD analyses	Glc (6), Gal (10), Xyl (12), Fuc (0), Ara (26), GlcA (0), GalA (0) / Glc (10), Gal (11), Xyl (14), Fuc (2), Ara (23), GlcA (2), GalA (2)		EPS	NA	
*Pavlova* sp. RCC 3438 ***Diacronema* sp.**	47 ^b^ GC/MS analyses / 30 ^b^ HPAEC-PAD analyses	Glc (11), Gal (27), Xyl (3), Man (trace), Ara (5), GlcA (5) / Glc (17), Gal (29), Xyl (6), Man (0), Ara (14), GlcA (4)		EPS	NA	
*Dunaliella* sp. RCC 5	18 ^b^ GC/MS analyses / 21 ^b^ HPAEC-PAD analyses	Glc (2), Man (11), Xyl (13), Gal (19), Ara (4), GlcA (19), GalA (14) / Glc (trace), Man (13), Xyl (17), Gal (28), Ara (5), GlcA (4), GalA (11)		EPS	NA	
*Tetraselmis globosa RCC 1564* ***Tetraselmis convolutae***	63 ^b^ GC/MS analyses / 61 ^b^ HPAEC-PAD analyses	Glc (5), Gal (19), Xyl (5), Ara (trace), GlcA (0), GalA (5) / Glc (5), Gal (20), Xyl (4), Ara (trace), GlcA (2), GalA (0)		EPS	NA	
*Klebsormidium flaccidum* 749B	20.9 ^d^	Ara (7.8), Fuc (12.4), Xyl (8.9), Man (37.8), Gal (7.4), Glc (4.8)	Uronic acids (9.3% weight) Sulfate (0% weight)	RPS	NA	[48]
*Klebsormidium flaccidum 446C*	49.3 ^d^	Ara (2.2), Fuc (3.9), Xyl (8.2), Man (31.5), Gal (3.4), Glc (1.5)	Uronic acids (15.0% weight) Sulfate (0.3% weight)	RPS	NA	
	31.9 ^d^	Ara (4.3), Fuc (10.1), Xyl (9.3), Man (27.1), Gal (10.4), Glc (7.1)	Uronic acids (8.7% weight) Sulfates (7.6% weight)	CPS	NA	
*Stichococcus bacillaris* 772B	37.8 ^d^	Ara (7.1), Fuc (1.0), Xyl (19.2), Man (12.0), Gal (17.3), Glc (5.6)	Uronic acids (14.6% weight) Sulfates (trace)	RPS	NA	
*Stichococcus bacillaris 774E*	27.4 ^d^	Ara (10.4), Fuc (5.4), Xyl (7.2), Man (16.1), Gal (22.0), Glc (11.5)	Uronic acids (12.0% weight) Sulfates (trace)	RPS	NA	
	35.6 ^d^	Ara (6.4), Fuc (2.8), Xyl (10.4), Man (8.2), Gal (19.4), Glc (17.1)	Uronic acids (9.4% weight) Sulfates (trace)	CPS	NA	
*Stichococcus bacillaris 747C*	32.2 ^d^	Ara (9.7), Fuc (2.6), Xyl (15.1), Man (14.4), Gal (19.8), Glc (6.2)	Uronic acids (20% weight) Sulfates (trace)	RPS	NA	
*Stichococcus bacillaris 677A*	33.2 ^d^	Ara (8.4), Fuc (2.0), Xyl (20.6), Man (11.1), Gal (20.7), Glc (4.0)	Uronic acids (11.6% weight) Sulfates (trace)	RPS	NA	
*Chlorella* cf. *mirabilis* 678F	31.6 ^d^	Ara (13.9), Fuc (2.6), Xyl (11.9), Man (15.2), Gal (14.2), Glc (10.7)	Uronic acids (14.1% weight) Sulfates (trace)	CPS	NA	
*Bracteacoccus* sp. 679E	23.0 ^d^	Ara (8.0), Fuc (5.2), Xyl (9.8), Man (19.6), Gal (18.1), Glc (16.4)	Uronic acids (10.3% weight) Sulfates (trace)	CPS	NA	
*Chlorella pyrenoidosa* ***Auxenochlorella pyrenoidosa***	34.7 ^b^	Man (12.0), GlcA (2.3), GalA (0.6), Glc (8.8), Gal (26.1), Ara (15.5)		EXP	NMR analysis	[93]
*Chlorella pyrenoidosa* ***Auxenochlorella pyrenoidosa***	17.1–37.8 ^a^	Man (8.5–15.2), Glc (12.7–18.1), Gal (26.7–46.5), Unknown (7.6–9.8)		EXP fractions	NA	[102]
*Chlorella pyrenoidosa* ***Auxenochlorella pyrenoidosa***	51.8 ^b^	Ara (12.2), Xyl (9.9), Man (4.5), Gal (13.1), GlcA (8.6) Galactose ratio of d- and l-enantiomorphs (73:27)		EXP	Study of partial acid hydrolysis products	[103]
*Chlorella pyrenoidosa* ***Auxenochlorella pyrenoidosa***	31.8%	Glc (20.4), Gal (10.3), Man (5.2), Xylose (1.3)		EXP	NA	[104]
*Desmodesmus armatus*	9.0–29.6 ^b^	Man (2.2–10.2), GlcA (2.5–12.7), GalA (1.5–7.2), Ara (4.3–46.9), Fuc (24.6–63.8)	Sulfates (0–0.56%) Uronic acids (11.80–18.20%)	EXP fractions	NA	[105]
*Navicula* sp.	10.67–35.34 g/100 g sulfated polysaccharide dry matter	Glc (15.46–29.23), Gal (16.82–24.48), Man (4.43–5.07), Xyl (5.18–10.38)	Sulfates (0.32–0.40 g/100 g sulfated polysaccharide dry matter)	EXP	NA	[106]
*Chaetoceros decipiens*	28 ^b^	Glc (4), Man (16), Xyl (4), Fuc (28), Gal (20)		EPS	Glycosidic linkage analysis using partially methylated alditol acetate derivatization	[107]

^a^: % weight/weight total sugar; ^b^: % mol/mol total sugar; ^d^: % mol/mol of total neutral sugars. EXP: extracted polysaccharides; EPS: exopolysaccharides; RPS: released polysaccharides; CPS: capsular polysaccharides; NA: not available; Rha: rhamnose; Gal: galactose; Glc: glucose; Xyl: xylose; GlcA: glucuronic acid; Man: mannose; Fuc: fucose; Ara: arabinose; GalA: galacturonic acid; Fru: fructose; Rib: ribose; N-GlcNAc: N-Acetyl-Glucosamine; N-GalNAc: N-Acetyl-Galactosamine; GlcN: glucosamine; GalN: galactosamine; NMR: Nuclear Magnetic Resonance; MS: Mass Spectrometry; ESI: Electrospray Ionization. Name in bold corresponds to the taxonomically accepted name according to AlgaeBase; https://www.algaebase.org/ (lastly accessed on 10 September 2026) [56] or from the Roscoff Collection Culture; https://www.roscoff-culture-collection.org/ (lastly accessed on 10 September 2026) [108] when it differs from the name used in the original article.

**Table 4:** Sources of rhamnolipids from marine bacteria.

Species	Rhamnolipids	References
Isolate M15 *Pseudomonas gessardii*	Rha-C8-C10 Rha-C9-C10 Rha-C12:1-C8 Rha-C10-C10 Rha-C11-C10 Rha-C12:1-C10 Rha-C12-C10 Rha-C12:1-C12:1 Rha-C14:1-C10 Rha-C12-C12:1 Rha-C12:1-C12 Rha-C14:1-C12:1 Rha-C14-C10 Rha-C16:1-C10 Rha-C14:1-C12 Rha-C12-C14:1 Rha-C16-C10	[112]
Isolate M10B774 *Pseudomonas fluorescence* group	Rha-C10-C10 Rha-C12:1-C10 Rha-C12:1-C10 Rha-C12-C10 Rha-C14:1-C10	[113]
Isolate MCTG107b *Marinobacter* sp.	Rha-C14:2 Rha-C10-C12/Rha-C12-C10 Rha-C10-C10:1 Rha-C12-C12/Rha-C10-C14 Rha-Rha-C8 Rha-Rha-C10 Rha-Rha-C14 Rha-Rha-C10-C10:1/Rha-Rha-C10:1-C10 Rha-Rha-C10-C10 Rha-Rha-C10-C12:1 Rha-Rha-C10-C10-CH3 Decenoyl-Rha-Rha-C10-C10:1 Rha-Rha-C10-C12/Rha-Rha-C12-C10 Rha-Rha-C10-C12-CH3/Rha-Rha-C12-C10-CH3	[114]
*actinomycete Streptomyces* sp. ISP2-49E	Rha-Rha-C10-C10	[115]
Isolate MCTG214(3b1) *Pseudomonas* sp.	Rha-Rha-C10 Rha-Rha-C10-C10 Rha-C10-C10/C8-C12 Rha-Rha-C10-C12/C12-C10 Rha-Rha-C8-C10/C10-C8	[116]
*Pseudomonas guguanensis*	ethyl 3-(((3*S,* 4*S,* 5*S,* 6*R*)-3,4,5-trihydroxy-6-methyltetrahydro-2H-pyran-2-yl)oxy) octadecanoate	[117]
*Marinobacter* sp.	Not available	[111]
*Pseudomonas mendocina*	Not available	

**Table 5:** Comparison of properties of the main monosaccharides found in algal polysaccharides.

Monosaccharide	Structural Information		LogP ^a^	Pka ^b^
l-Rhamnose	Aldose	Deoxyhexose	−2.1	12.46
l-Fucose			−2.1	12.50
d-Galacturonic acid		Uronic acid	−2.3	3.48
d-Glucuronic acid			−2.3	3.20
l-Arabinose		Pentose	−2.5	12.34
d-Ribose			−2.5	12.46
d-Xylose			−2.5	12.15
d-Galactose		Hexose	−2.6	12.35
d-Glucose			−2.6	12.28
d-Mannose			−2.6	12.08
d-Fructose	Ketose	Hexose	−2.8	12.03

^a^ Data from https://pubchem.ncbi.nlm.nih.gov (lastly accessed on 10 September 2026), ^b^ [178].

## Data Availability

No data supporting reported results will be shared excepting. The authors can be contacted to get details and datasets, according to privacy or legal restrictions from ANR.

## Abbreviations

The following abbreviations are used in this manuscript:

Ara	arabinose
CID	collision-induced dissociation
CPC	centrifugal partition chromatography
CPS	capsular polysaccharides
DEAE	diethylaminoethyl
dTDP	deoxythymidine diphosphate
dTDP-rhamnose	deoxythymidine 5′-diphospho-β-l-rhamnose
EPS	exopolysaccharides
ESI	electrospray ionization
EXP	extracted polysaccharides
Fru	fructose
Fuc	fucose
Gal	galactose
GalA	galacturonic acid
GalN	galactosamine
GH	glycoside hydrolase
Glc	glucose
GlcA	glucuronic acid
GlcN	glucosamine
GT	glycosyltransferase
GulA	guluronic acid
IdoA	iduronic acid
MALDI-TOF	matrix-assisted laser desorption/ionization time-of-flight
Man	mannose
ManA	mannuronic acid
MS	mass spectrometry
NA	not available
NAD	nicotinamide adenine dinucleotide
NADP	nicotinamide adenine dinucleotide phosphate
N-GlcNAc	N-acetyl-Glucosamine
N-GalNAc	N-acetyl-Galactosamine
NMR	nuclear magnetic resonance
PL	polysaccharide lyase
PUL	polysaccharide utilization loci
Rha	rhamnose
RHM	rhamnose synthase
Rib	ribose
RPS	released polysaccharides
TFA	trifluoracetic acid
Xyl	xylose
UDP	uridine diphosphate
UDP-rhamnose	uridine 5′-diphospho-β-l-rhamnose
